# Data selection strategies for minimizing measurement time in materials characterization

**DOI:** 10.1038/s41598-025-96221-1

**Published:** 2025-04-30

**Authors:** Alexander Liehr, Kristina Dingel, Daniel Kottke, Sebastian Degener, David Meier, Bernhard Sick, Thomas Niendorf

**Affiliations:** 1 Institute of Materials Engineering, University of Kassel, Moenchebergstr. 3, 34125 Kassel, Germany; 2 Intelligent Embedded Systems, University of Kassel, Wilhelmshöher Allee 71-73, 34121 Kassel, Germany; 3 Bundesanstalt für Materialforschung und -prüfung, Unter den Eichen 87, 12205 Berlin, Germany; 4 Helmholtz-Zentrum für Materialien und Energie, Hahn-Meitner-Platz 1, 14109 Berlin, Germany

**Keywords:** Characterization and analytical techniques, Metals and alloys, Mechanical engineering

## Abstract

Every new material needs to be assessed and qualified for an envisaged application. A steadily increasing number of new alloys, designed to address challenges in terms of reliability and sustainability, poses significant demands on well-known analysis methods in terms of their efficiency, e.g., in X-ray diffraction analysis. Particularly in laboratory measurements, where the intensities in diffraction experiments tend to be low, a possibility to adapt the exposure time to the prevailing boundary conditions, i.e., the investigated microstructure, is seen to be a very effective approach. The counting time is decisive for, e.g., complex texture, phase, and residual stress measurements. Traditionally, more measurement points and, thus, longer data collection times lead to more accurate information. Here, too short counting times result in poor signal-to-background ratios and dominant signal noise, respectively, rendering subsequent evaluation more difficult or even impossible. Then, it is necessary to repeat experiments with adjusted, usually significantly longer counting time. To prevent redundant measurements, it is state-of-the-art to always consider the entire measurement range, regardless of whether the investigated points are relevant and contribute to the subsequent materials characterization, respectively. Obviously, this kind of approach is extremely time-consuming and, eventually, not efficient. The present study highlights that specific selection strategies, taking into account the prevailing microstructure of the alloy in focus, can decrease counting times in X-ray energy dispersive diffraction experiments without any detrimental effect on data quality for the subsequent analysis. All relevant data, including the code, are carefully assessed and will be the basis for a widely adapted strategy enabling efficient measurements not only in lab environments but also in large-scale facilities.

## Introduction

It is well-known that the microstructure of any material dominates its macroscopic behavior. In addition to its basic structure, the periodicity of the arrangement of atoms, preferred grain orientation, residual stress fields, and local phase composition play an essential role in terms of the strength and lifetime of real components. Especially for reducing the emission of $CO_{2}$, advanced alloys need to be developed and analyzed. Here, promising candidates are lightweight components, e.g., made of high-strength steel. In a number of novel steel concepts, the stress- and strain-induced transformation of metastable austenite into martensite is an essential factor contributing to the final mechanical properties under a given external load. This transformation promotes local hardening and, eventually, leads to a delay of necking and overload failure. Thus, the interplay of the elementary deformation mechanisms prevailing in these steels contributes to high ductility and high tensile strength simultaneously, known as transformation-induced plasticity (TRIP) effect^1^. Quench and Partitioning (QP) steels represent the most recent generation of high-strength steels combining high strength and good ductility. The concept is essentially based on two steps, i.e., quenching to set a defined martensite content, followed by a heat treatment step to stabilize the retained austenite through targeted carbon partitioning between martensite and austenite. A predominantly martensitic structure with retained austenite and minor fractions of bainite/ferrite is obtained after the QP heat treatment. Due to their outstanding mechanical properties at comparatively low costs, QP steels are of particular interest to the automotive industry, where high strength in combination with sufficient ductility and low component weight are desired to enable maximum safety under the consideration of maximum resource efficiency. Another advantage is the ease of integration of the heat treatment strategy into existing production lines, making the use of QP steels even more sustainable^2^. It has already been discussed that the complex microstructures and material behavior, respectively, require a tremendous increase in characterization efforts to fully understand the corresponding properties and derive process-microstructure-property relationships^3^. This understanding will also promote further advances in additive manufacturing of microstructurally graded TRIP steels and other metallic alloys, where only an increase in efficiency of the applied methods will allow the intended direct design of form and function^4^.

High-resolution strain or texture mappings using X-ray diffraction (XRD), local hardness tests, non-destructive analysis of steep residual stress gradients by energy dispersive XRD, and *in situ* stress or phase analysis, i.e., conditions probed under external mechanical load, represent possible analytic methods^5,6^.

Because of the wide range of different microstructures in novel high-strength materials, there is great interest in the availability of fast, phase-selective, reliable, and non-destructive methods such as XRD-enabled assessments. Considering diffraction techniques, analysis of the crystallographic structure of a crystalline material, its crystallographic orientation, and the phase content is feasible^7^. Based on specific scanning strategies, texture and lattice strain measurements can be conducted. Here, one possibility is to analyze the maximum intensity of a prevailing interference peak with respect to the sample orientation; another approach is to determine the lattice strains based on the exact position of the peak, from which the residual stress states are calculated using elastic theory and suitable elastic constants^7^. When using such strategies, sufficiently accurate peak profile analysis is necessary to obtain reliable results. Depth-resolved measurements can be conducted either by angle dispersive or energy dispersive XRD methods to assess the properties of the surface regions^5^. Here, for the angle dispersive approach layer removal is needed, eventually increasing the efforts needed for assessing in-depth profiles.

Novel materials like QP steels, as well as novel manufacturing processes like additive manufacturing, are often characterized by the presence of graded sample conditions. Complex and time-consuming XRD mapping strategies are then applied to probe local measurement positions across given component regions. Here, an online data refinement would be highly beneficial to realize laboratory measurements without investing too much experimental time, while still providing sufficient exposure time to achieve sufficient signal intensity.

Breidenstein et al.^6^ performed an analysis, where they tried to identify a sufficient exposure time based on an asymptotic approximation, taking into account data scatter. Here, a complex series of measurements and follow-up evaluations had to be carried out to find a suitable exposure time for the material under examination. This strategy is rather time-consuming and requires repetition for each new alloy under investigation. The results of the mentioned study point to the fact that constantly repeating evaluation steps, which only serve as a simple observation of defined target variables, do not always have to be carried out by the operator himself. Typically, such target variable observations are carried out after an experimental phase, where the experiment often is even conducted with not optimally selected exposure times.

Intelligent experimental design intends to counteract this by integrating data evaluation procedures into the experimentation process, also known as closed-loop experimentation. The field of materials science experimentation is currently witnessing a surge in research efforts toward this direction^8–11^. Active learning strategies, in particular, have shown to be very effective for closing the experimentation loop and saving experimentation time by querying the most promising sample next^12^. Yet, many other techniques such as statistical inference^13^, sequential learning^14^, co-design approaches^15^, or uncertainties from machine learning models^16^ can be used to enhance experimentation. There are hardly any limits for applying intelligent experimentation, e.g., using machine learning for additive manufacturing^17^, alloy deployment^18^, or X-ray scattering^19^. Especially deep learning methods^20^, due to their rapid inference capabilities, harbor great potential to advance several aspects of experimentation.

In the present article, we aim to demonstrate the effectiveness of integrating immediate data evaluation into the experimental process using the application case of XRD-based retained austenite analysis. Utilizing the data collected up to a specific experimentation iteration, we demonstrate how different selection strategies in terms of the energy-interval in the experimentally determined spectra affect the next experiment iterations and the required time to accurately characterize key intensity peak characteristics, namely the position of the diffraction line, peak height, peak area, and integral width. The experiments were conducted using a low alloy 42CrSi QP steel. Precisely, we compare the state-of-the-art so far, namely sequential acquisition, with two new and more intelligent selection strategies, where we (1) use the regions-of-interest (ROI) within the energy range or (2) investigate the next relevant energy interval using the minimum volume of the intensity peaks so far acquired. Using these selection strategies and extending a recent article^21^, we endeavor to provide preliminary answers to the following questions:

- How can we intelligently select exposure times and energy ranges, respectively, for investigation?
- When is it appropriate to terminate an ongoing experiment?

To shed light on the procedures applied, we introduce the utilized materials and methods, discuss results and advantages when using the proposed data selection strategies, and provide an outlook on the effects these strategies will have for energy-dispersive XRD experimentation in general.

## Materials and methods

### High strength QP steel with metastable retained austenite

In the present work, we used a low-alloy 42CrSi steel to study a complex microstructure, including a metastable phase. In a previous study, we already investigated and characterized the material in depth by means of *in situ* XRD experiments^3^. There, it was revealed that laboratory-based XRD methods show high potential for reliable assessment, but rather are time-consuming. In follow-up work, we could reveal that a higher retained austenite content promotes an increased fatigue strength. In all cases, assessment of localized phenomena was the key to finally rationalize global properties and, thus, provide statements on the integrity and reliability of the component. These examples point to the important role of local measurements, i.a., to assess the stability of the retained austenite and the phase-selective residual stress states after or during application of an external load.

**Figure 1:**
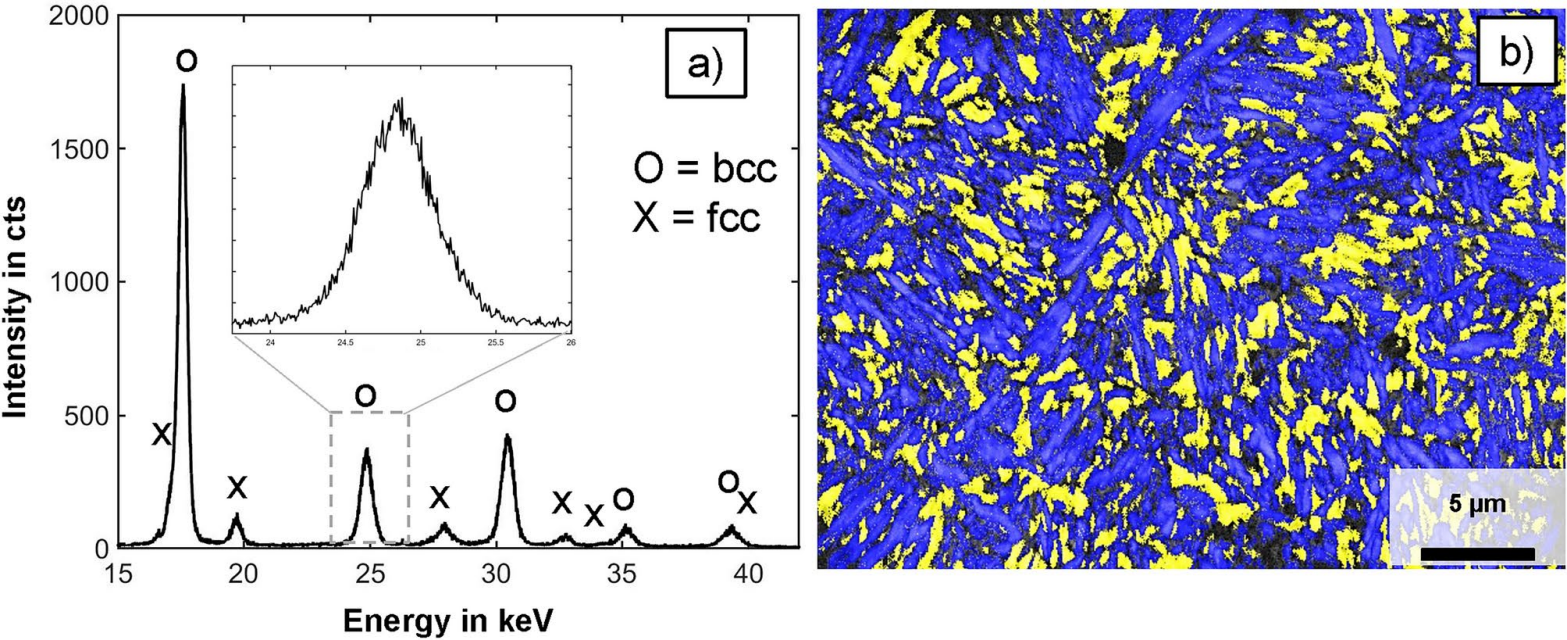
(**a**) Integrated spectra profile from 42CrSi steel with austenite and ferrite peaks obtained using a white X-ray beam of a tungsten tube and an exposure time of 15 times 900 sec during a diffraction experiment at one specific measurement point. The peak positions from the austenitic phase are marked with an “x”; ferrite/martensite peaks are marked with an “o”, (**b**) EBSD phase map superimposed to the quality map of the same sample with the retained austenite colored in yellow and the ferrite/martensite in blue. Recompiled from^3^.

A miniature sample with the dimension of 8 $\times$ 8 $\times$ 25 mm$^{3}$ was austenitized at a temperature of 950 $^\circ$C, quenched to 170 $^\circ$C in liquid salt, and – before cooling down to room temperature – a partitioning procedure was carried out at 400 $^\circ$C for 10 min. Afterward, dog-bone-shaped tensile test samples with gauge section dimensions of 18 $\times$ 3 $\times$ 1 mm$^{3}$ were manufactured by electrical discharge machining (EDM). Figure 1a shows the summed spectra (15 $\times$ 900 sec exposure time) highlighting the analyzed diffraction peaks of the body-centered cubic (bcc) ferrite and the face-centered cubic (fcc) austenite. The inset shows a detailed view of a peak of the 200-lattice plane from the ferrite phase. The electron back-scattered diffraction (EBSD) image in Fig. 1b shows the needle-like appearance of the retained austenite, colored in yellow, in a matrix of ferrite, colored in blue. The effort to experimentally determine such a spectrum is described in more detail in the following.

The dog-bone-shaped sample was loaded for the *in situ* test using a Kammrath and Weiss stress rig; for details, see^3^. Preliminary XRD investigations revealed that for this specific condition of the QP steel, the RA content decreases from around 14% within the clamping section to around 2.5% at the point of fracture.

### Physical basics of energy-dispersive X-ray diffraction

Applying XRD methods to characterize the microstructure of crystalline materials is common in the field of non-destructive methods. Here, it is feasible to study the stability and/or the change of microstructures and residual stress states, depending on thermal and mechanical loads. These experiments are based on the diffraction of X-rays within the atomic structure of crystalline materials described by the Miller indices, also known as *hkl* lattice planes. The correlation between the constructive interference of the diffracted X-rays, characterized by the used wavelength $\lambda$, under a specific angle $\theta$ with the distance *D* of the studied *hkl* lattice planes (being characteristic for the probed material) is described by Bragg’s law:

1 $$\begin{aligned} n\lambda = 2D \sin {\theta }. \end{aligned}$$

**Figure 2:**
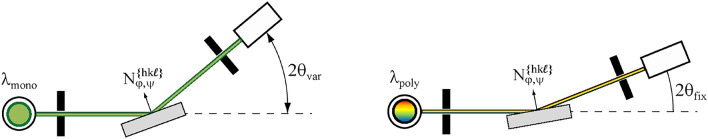
Schematic principle of the angle-dispersive approach with a constant beam energy of 6.93 keV of Co K$\alpha$ radiation (left) and the energy-dispersive measurement mode with an applicable energy range (white beam) from 1 to 60 keV (right)^5^.

Every crystalline structure, represented by the stacking order of the atoms and the corresponding *hkl* lattice planes, has its own specific diffraction line profile. This can be rationalized by the fact that Bragg’s law is only fulfilled in specific positions corresponding to these atomic positions, eventually related to the distance *D* of the atomic lattice planes. If *D* needs to be determined experimentally for assessment, Eq. 1 allows for two different strategies. Either $\lambda$ is kept constant, while the integer multiple *n* is equal to one and $\theta$ needs to be determined experimentally, or $\theta$ is constant, and $\lambda$ will be considered in the experiment. In both cases, the diffracted intensity is in focus. In laboratory applications, the angle dispersive mode is preferred. The wavelength $\lambda$ of the X-ray tube is fixed, whereas the diffraction angle $\theta$ is varied to scan the intensity of a peak profile step-wise and calculate the exact angle $\theta$ for the corresponding value *D* of the *hkl*-lattice plane in focus afterward. This procedure is schematically shown in the left part of Fig.  2. In the energy dispersive mode, the diffraction angle $\theta$ is fixed and a variable primary wavelength (white spectrum) is analyzed continuously by an energy-resolved semiconductor detector. In this case, the complete spectrum of the used X-ray source, the so-called white beam linked to the *Bremsstrahlung*, can be used and a step-wise scanning procedure is not necessary^5^. This strategy is shown in Fig. 2 to the right.

In both approaches, the focus is on analyzing the characteristics of the peaks for different *hkl* diffraction lines, e.g., the peak width, integral area, height, and exact position. The main target of the specific analysis strategy has always to be considered at this point. In the energy-dispersive mode, the information of the spectral profile is increasing continuously for every prevailing *hkl* peak. At least under laboratory conditions, the scan profile in the angle dispersive mode follows a step-wise approach with a constant exposure time per step. To analyze a full interference profile, it is necessary to finish a scan before starting the next step in the procedure. Both methods can be applied in reflection geometry (incident X-ray beam diffracted in the surface area of the sample) and transmission geometry (incident X-ray beam penetrates a relatively thin sample). The reflection mode is the common use case for laboratory conditions. In the case of an XRD mapping, a fixed $2\theta$ angle is a beneficial boundary condition. This is due to the fact that the shape of the illuminated spot on the sample surface remains constant. The constantly growing spectrum during an energy dispersive measurement enables the use of the already collected peak profile data from an energy interval $\Delta \lambda$ to intelligently choose the next exposure time. This can be done continuously, while the quality measures focus on accurately analyzing the specific peak characteristics. If the quality is good enough, the running measurement sequence can be stopped immediately without losing too much time at this measuring point (MP). This is only possible in the case of continuous data recording and, thus, the energy dispersive approach is excellently suited at this point. The focus of the present study is not on the quality standards of the energy dispersive structure, stress, phase, or texture analysis, but rather on pathways to reduce the scanning time, eventually enabling faster measurements without losing necessary information. It is shown that by simply implementing different data selection strategies into the measurement script, intelligent closed-loop experimentation becomes feasible.

### Setup and data processing

**Figure 3:**
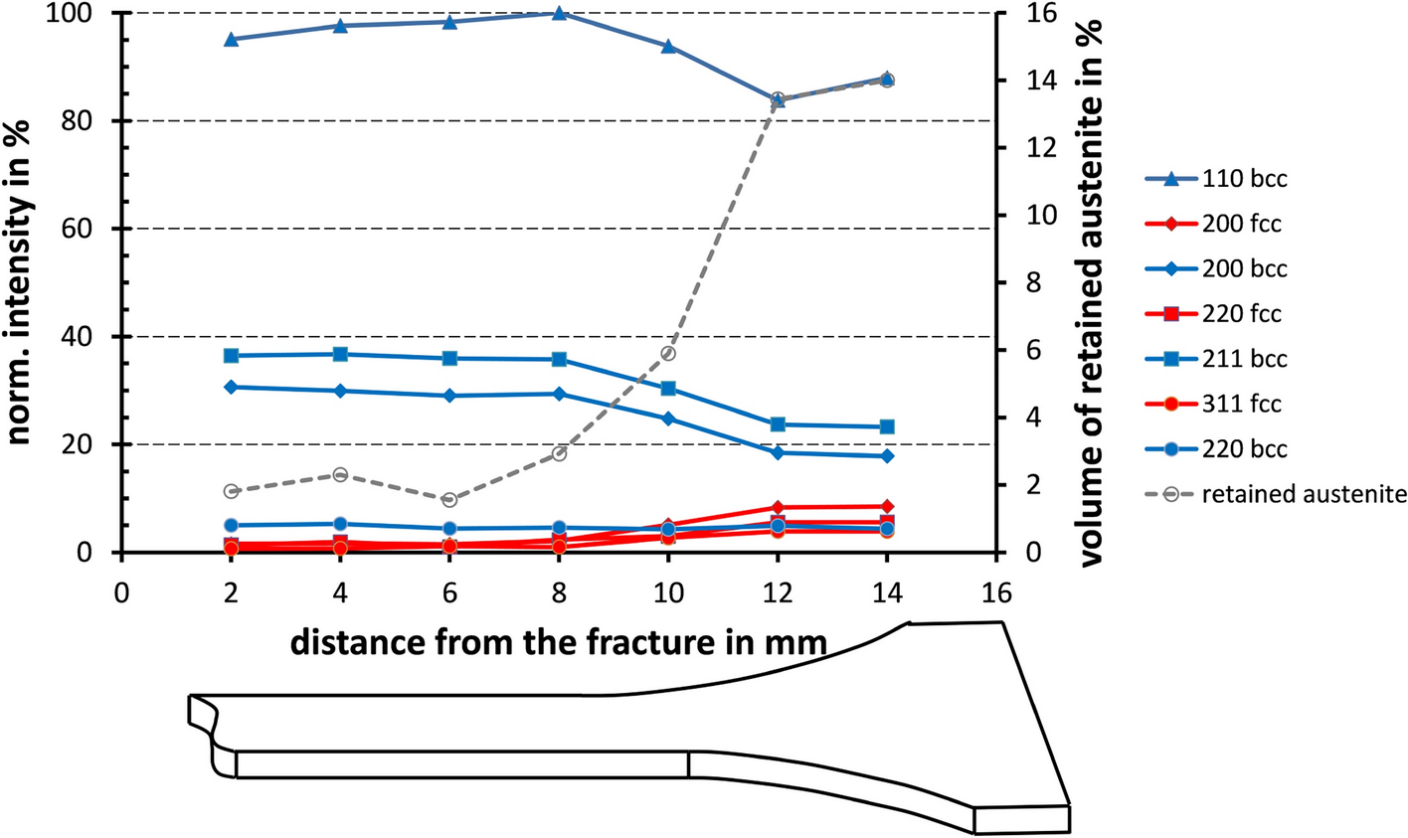
Intensities of the *hkl* lattice planes normalized to the 110 lattice plane (left axis) and estimated volume of the retained austenite content (right axis) plotted as a function of the corresponding measurement position. The sketched sample is shown below the graph schematically.

The measurements were performed on an energy-resolved X-ray diffractometer using the *Bremsspektrum* of a conventional laboratory X-ray source with tungsten anode^22^. The white beam was generated with an acceleration voltage of 60 kV and a current of 40 mA and collimated by a 0.5 mm pinhole collimator with a length of 200 mm. The X-ray tube and the Si-drift detector AXAS-M of KETEK were positioned symmetrically at a distance of 340 mm to the sample with a diffraction angle $2\theta$ of 20$^\circ$. In the present work, we used a dog-bone-shaped tensile sample of the QP steel detailed before. This sample was probed by a line scan with a step size of 2 mm, starting close to the fracture region (MP1 = 0 mm) and ending within the clamping area (MP8 = 14 mm). Using this strategy, eight measurement points were analyzed along the sample. Continuous spectra were acquired for 4 h at each of these eight MPs at the sample surface, as highlighted on the X-axis in Fig. 3. An exemplary spectrum of measurement point 6, i.e., at a position 10 mm away from the crack surface of the sample (cf Fig. 3), is depicted in Fig. 1a. Data of each spectrum are saved in a two-column table file containing detector channels (first column) and intensity (second column [absolute counts]) with a preceding five-line header. For further data processing, we converted the detector channels to energies using an earlier recorded energy calibration function. We used a measurement on a certified ENSAM Fe powder (with the same measurement setup) to elaborate the calibration function. In the experiment shown here, the energy-resolved intensity of each spectrum increases over 900 s. This corresponds to 16 individual spectra for each of these 8 MPs. According to this procedure, 128 spectra were available for further data processing in the form of text files (intensity in absolute counts over energy in keV), exemplarily depicted in Fig. 1a. Table 1 provides an overview of all available data. To analyze the phase fractions or the retained austenite (RA) content in the eight MPs, the areas of the phase-specific peaks are weighted and evaluated following the strategy detailed in^3^. Figure 3 shows the normalized peak areas in relation to the largest peak and the volume fraction of RA determined from the data, for each of the eight probed MPs.

In the specific MPs probed here, the RA content varies from a maximum volume content of 14 % to a minimum of around 2%, in line with the results already published^3^. The first measuring point (MP1 position 0 mm) could not be clearly evaluated due to the fact that severe plastic deformation occured in direct vicinity of the fracture surface.

**Table 1:** Overview of the available data corresponding to the plotted results shown in Fig. 3.

Measurement point (.txt file)	Position [mm]	# Spectra	Measurement Duration / Spectrum [s]
mcaData_MP1_combined.txt	0	16	900
mcaData_MP2_combined.txt	2	16	900
mcaData_MP3_combined.txt	4	16	900
mcaData_MP4_combined.txt	6	16	900
mcaData_MP5_combined.txt	8	16	900
mcaData_MP6_combined.txt	10	16	900
mcaData_MP7_combined.txt	12	16	900
mcaData_MP8_combined.txt	14	16	900

### Simulation of *in situ* experiments

The major goal of the present study is to determine the potential of the approach for reducing the time necessary to perform an experiment that captures the peak width *w*, the integral area *a*, the peak height *h*, and the exact peak position *p* of Bragg peaks. The main idea is to focus on peaks that are not entirely evident in these characteristics and select specific energy intervals to increase accuracy concerning the Bragg peaks.

As evaluating such a strategy in an *in situ* experiment is time-consuming and expensive, we decided to simulate the experimentation process using the aforementioned data. Algorithm 1 schematically shows the steps that need to be performed during experimentation. This procedure is straightforwardly transferable to a real experiment. The individual steps will be discussed in more detail below.

**Algorithm 1:**
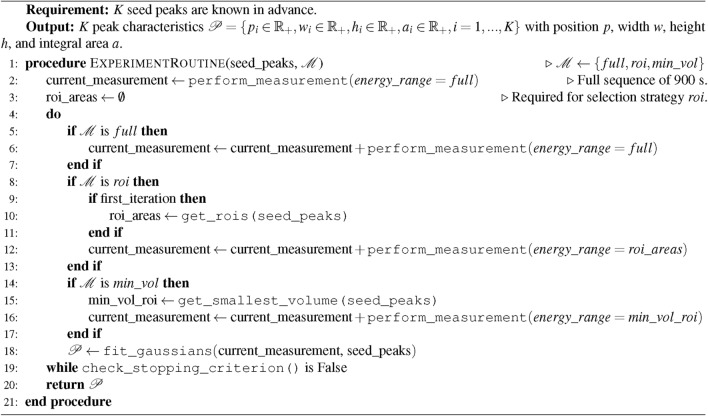
Overview of the simulated energy dispersive XRD experimentation procedure using multiple selection strategies.

**Figure 4:**
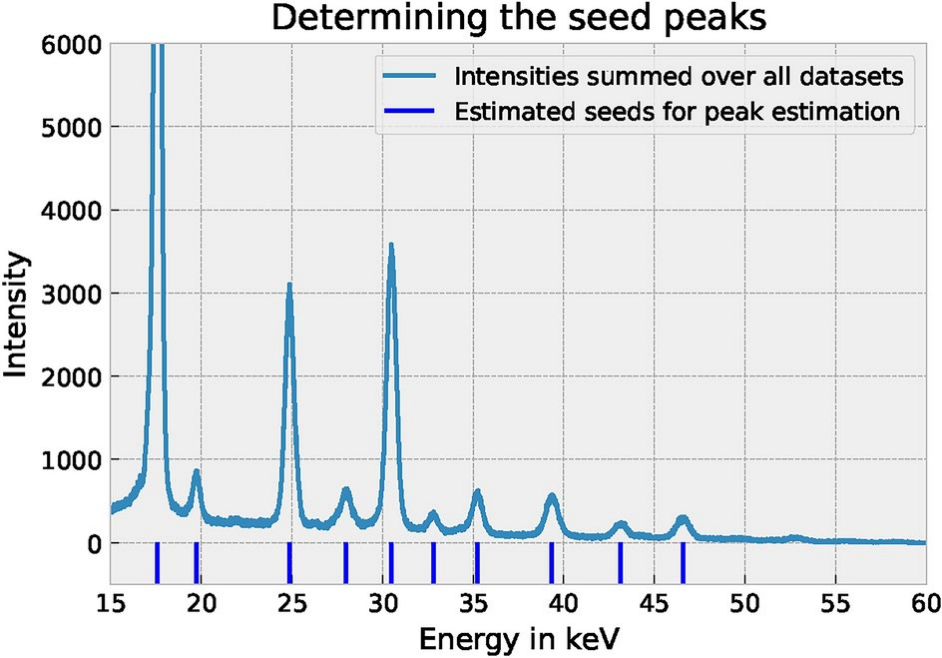
Identified seed positions for the Bragg peaks based on the specific spectrum of the QP steel, additionally provided in Table 2.

**Identifying seed peaks:** We identified so-called seed positions $\hat{E}_i$ for the Bragg peaks based on the material properties given in Table 2. Every seed position defines an interval $I_i = [\hat{E}_i - \frac{w}{2}, \hat{E}_i + \frac{w}{2}]$, with a pre-defined width parameter $w = 3~\textrm{keV}$. To find the seed peaks, we averaged all measurements (4 hours, 8 experiments). After smoothing the curves further, we used the first and second derivatives to identify the maximum value in that smoothed curve. Next, we identified the number of peaks that we want to consider (here 10) and used the extracted maximum values as the seed peaks for further investigation. It must be emphasized that the seed peaks 1 and 9 (cf. Table 2) consist of a superposition of two Bragg peaks. Evaluation strategies are known, in which this superposition can also be separated, however, this is not the focus of the present study. The results elaborated here are provided in Fig. 4. Clearly, these seed peaks are not accurate, but they will be refined in the upcoming experiments.

**Table 2:** Crystal structure *hkl* lattice planes and $\hat{E}_i$ seed positions for Bragg peaks used in the experiments.

*i*	1	2	3	4	5	6	7	8	9	10
*hkl*	111+110	200	200	220	211	311	220	400	331+222	321
*structure*	fcc+bcc	fcc	bcc	fcc	bcc	fcc	bcc	fcc	fcc+bcc	bcc
$\hat{E}_i$ [keV]	17.602	19.738	24.881	27.992	30.498	32.81	35.235	39.351	43.119	46.573

**Figure 5:**
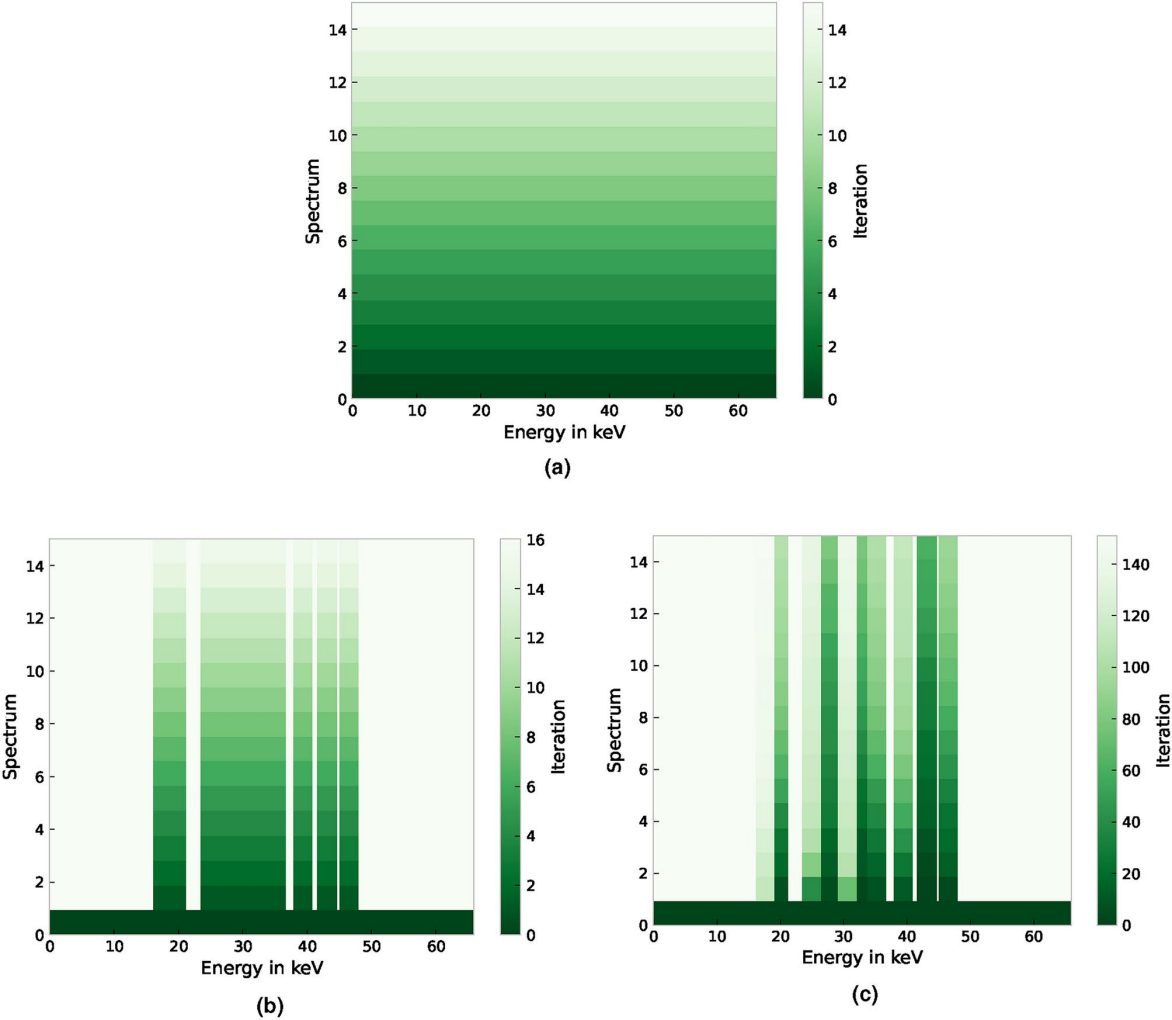
Visualization of all investigated selection strategies (**a**) *full*, (**b**) *roi*, and (**c**) *min_vol* for the dataset mcaData_MP6_combined.txt. The iteration determines the order of the performed measurements for the exemplary used QP steel.

**Initializing the experiment:** Regardless of the chosen selection strategy, the initialization of each experiment is done by measuring 900 s throughout the whole energy range (iteration 0 in Fig. 5). Then, a selection strategy decides which energy interval is to be probed next. To simplify the comparison, we only allow the selection of an energy interval or the whole energy range. If one interval is selected, we add the intensities of the selected interval (for the consecutive measurement period) from the original experiment. As, in the present study, the whole measurement was 4 h with a period time of 900 s, we have 16 periods to get data from. By normalizing the measured intensities by time for every energy level, we yield comparable characteristics independent from the total measurement time.

**Determining peak characteristics:** To determine the peak width *w*, the integral area *a*, the peak height *h*, and the exact peak position *p*, we fit a Gaussian function with height $\hat{h}$ and bias $\hat{b}$ parameters as given in Eq. 2 using a non-linear least-squares optimizer.

2 $$\begin{aligned} f(x) = \hat{h} \cdot \exp \left( -\frac{(x-\mu )^2}{2 \cdot \sigma ^2}\right) + \hat{b} \end{aligned}$$

We use the intensities measured over the complete time of 4 hours to identify the ground truth information. Based on the seed peaks, we fit the function in Eq. 2 to describe the target position $\mu ^*$, variance $\sigma ^*$, height $h^*$, and bias $b^*$ for every peak. For each of the eight measurement points, we provide the results of this estimation method in Supplementary Figures 1–3 (Appendix A).

**Selection strategy requirements:** To estimate the peak parameter, we assume to be able to acquire intensities for different intervals $[\hat{E}_l, \hat{E}_r]$ from each of the 16 $\times$ 900 s measurements. All methods start with one full sequence of 900 s. Then, each selection strategy successively chooses a specific interval (to acquire the data from) for the other 15 measurements.

**Selection strategy** ***full***: **Sequential acquisition** This method builds the baseline, i.e., the state-of-the-art so far. It consists of capturing data for the whole energy range [1 keV, 60 keV]. As each measurement in our experiment is divided into 16 blocks of 900 s length, the whole measurement period takes 4 h, eventually consisting of 16 estimates that should improve with more time. A visualization of the selection strategy can be seen in Fig. 5a.

**Selection strategy** ***roi***: **Sequential ROI acquisition** Knowing seed positions, we can omit all measurements that are probably not helping when estimating the peak parameters. Therefore, we use the above-mentioned energy intervals $I_i$ as regions of interest (ROI). In this baseline, we ask for all intervals in one of the 900 s measurements at once. Hence, determining the peak parameters should be faster compared to sequential acquisition without reducing their accuracy. A visualization of the selection strategy can be found in Fig. 5b.

**Selection strategy** ***min_vol***: **Minimum volume acquisition** While using the intensities per measurement time to fit the peak parameters, we use simple (non-normalized) intensity counts for this selection strategy. Our main assumption is that the reliability of the estimated parameters is strongly affected by and correlated with the measurement noise. While characteristics from peaks with large volumes can be reliably estimated, small peaks are much more difficult to characterize. Hence, we propose to select the peak with the smallest (non-normalized) intensity and volume, respectively, for the next measurement. Note that we usually normalize the data by the local (with respect to the energies) measurement time. If we would do this here, we would only select the smallest peak. Instead, we want to select the peak with the lowest intensities. Hence, we use the non-normalized volume. A visualization of the selection strategy can be found in Fig. 5c.

**Stopping the experiment:** In the present study, the stopping criterion is defined by the limitation of available data, namely 16 $\times$ 900 s measurements per file. This means that data acquisition stops if all available data have been requested. Therefore, the last iteration in each data selection strategy consists of requesting all remaining data as there is no further informative energy interval.

## Results

**Figure 6:**
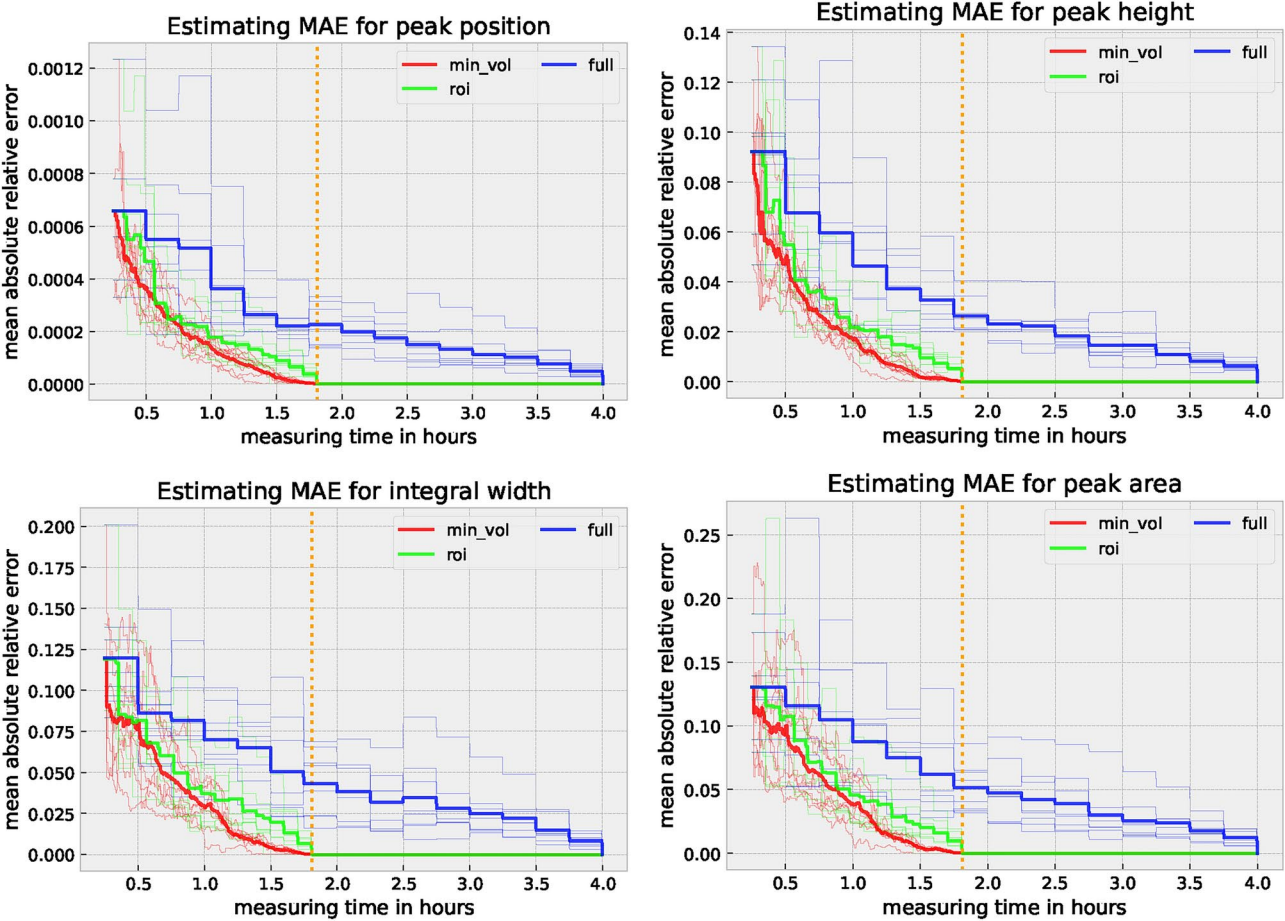
Relative error averaged over all estimated peaks (12 peaks in total) for every dataset (plus mean in bold) with respect to the measuring time. 1.81 hours (orange dotted line) of measurement time would be enough to reach an MAE of 0.0 according to the selection strategies *roi* and *min_vol*.

In this experiment, we exemplary use a dual-phase material with locally different contents of retained austenite, i.e., the low-alloy 42CrSi QP steel, and investigate the estimation error of each parameter over time for every selection strategy. It is important to mention at this point that this procedure generally applies to any other material of interest. In Fig. 6, we use the mean absolute relative error (MAE) across all relevant peak values (peak position *p*, integral width *w*, peak height *h*, and integral area *a*) for every phase-specific spectrum line (thin lines) and present their average (bold line).

### Best strategy

To compare the performance of the strategies, we use all available measurement data to determine the Bragg peak characteristics *p*, *w*, *h*, and *a*, which we assume to be the ground truth. Therefore, all selection strategies converge to zero error after the whole energy range for every measurement period has been acquired. This convergence is used for validation and allows for giving an estimate of how well each selection strategy performs. In practical applications, convergence to the smallest possible R$^{2}$ values or against user-specific limits would be necessary. These can vary depending on the application. For example, the accuracy of the peak position *p* is key for residual stress measurements, while the peak height *h* is most important during texture measurements.

For the selection strategy *full*, we see an MAE drop every 15 minutes (= 900 s); for *roi*, this MAE drop is every $\approx$ 400 s as the non-informative energy ranges are excluded by only investigating the energy intervals enveloping every seed peak. In the *min_vol* selection strategy, we can ask for each peak individually. Hence, the adaptability is improved compared to the strategy *roi*. Especially for spatially resolved residual stress, phase, or texture analysis, the *min_vol* strategy can be of great potential since a diffraction peak with the lowest intensity in one spectrum does not necessarily have the lowest intensity in another spectrum. With each interpolation point of a mapping on a component or sample surface to be examined, this time advantage will even be multiplied. This is also confirmed by the following observation from the results plotted in Fig. 6. The average MAE (bold line) when using the *min_vol* selection strategy is always below *full* and mostly below *roi*. Especially the *min_vol* selection strategy is suitable to stop even before reaching the MAE 0.0 mark (orange line in Fig. 6) in case a suitable stopping criterion, such as a predefined MAE threshold, is provided.

### Time-saving possibilities

**Figure 7:**
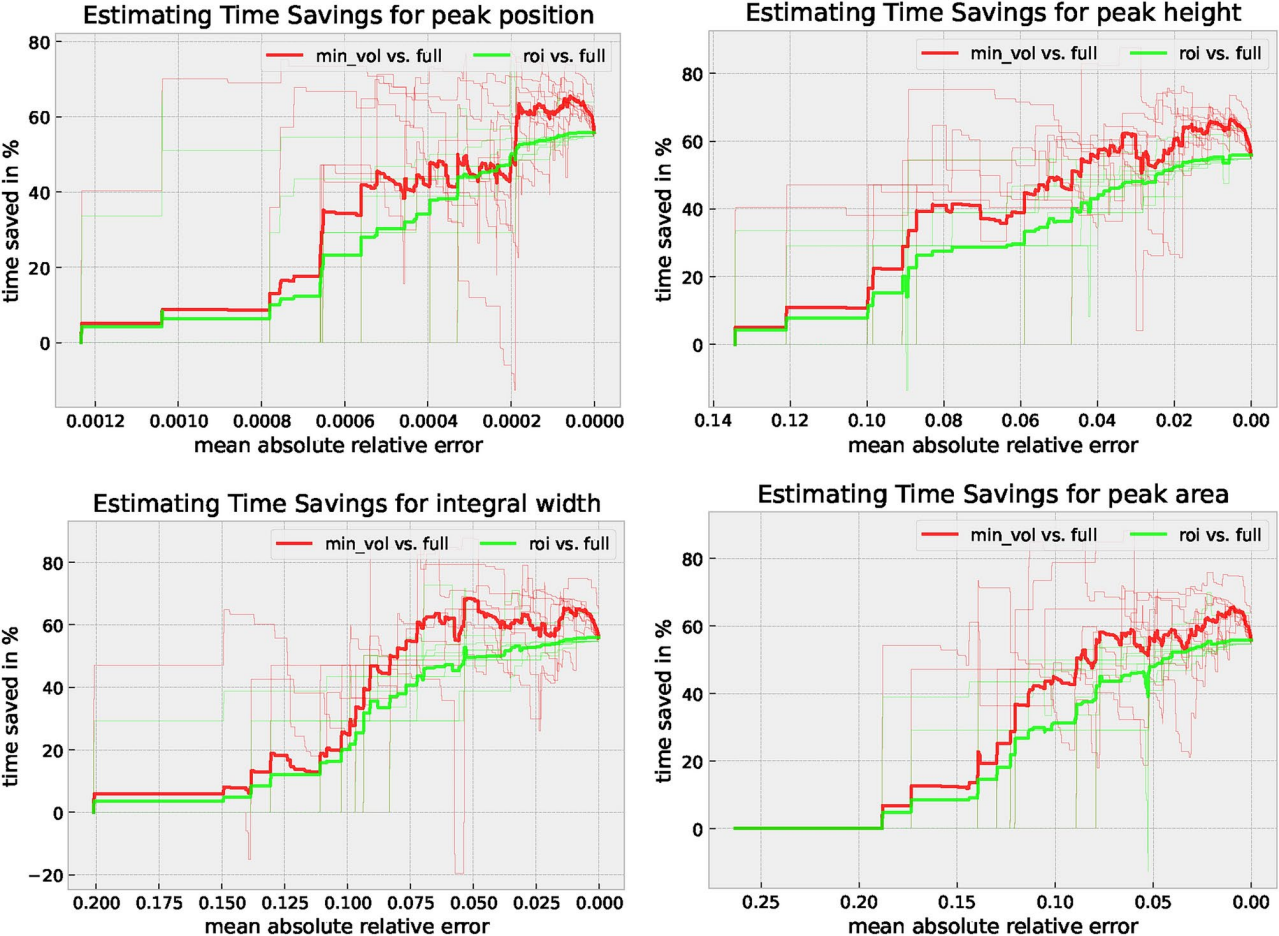
Relative error averaged over all estimated peaks (12 peaks in total) for every dataset (plus mean in bold) with respect to the measuring time.

Figure 7 shows the potential time savings with respect to the acceptable MAE. This relationship shows that the saved time is up to 62% for *min_vol* and up to 55% for *roi*. We also see the benefits of intelligently selecting the intervals using *min_vol*. Still, it needs to be considered that some peaks of specific phases are only found at times below the baseline. Here, the curve fitting algorithm was not robust enough and the peak was not successfully detected. This occurs especially in case of measurements close to the resolution limit of the setup, which in some cases can still be compensated by smoothing the background noise. In some cases, such an erroneous peak fit must be removed from the subsequent evaluation chain. In the case considered here, values falling below the baseline could be interpreted as a quality feature, which can already be evaluated or classified by an algorithm during a measurement. This would have the consequence that this peak is either excluded from the following data processing, or the specific counting time is extended until this effect no longer appears.

Especially for continuous measurements like those considered here, i.e., using an energy dispersive setup or, in the case of a line or area detector, the integration of an online data evaluation component is of extreme advantage. Since complex measurement sequences are often created by means of programmed scripts in large research facilities and also laboratory devices, the integration of the *min_vol* strategy is quite promising, especially since the subsequent process of data processing is often a standardized procedure. In some cases, large amounts of experimental data are first generated in the *TeraByte* range and then analyzed step by step in a time-consuming process. Particularly in the case of studies conducted at large research facilities, this means that the time required for data analysis is significantly higher than the time required for data acquisition.

## Discussion and outlook

In the present study, we investigated different selection strategies to perform faster XRD measurements without reducing accuracy. We used experimentally determined, periodically measured diffraction profiles as a basis for simulating a real-time measurement and integrated the selection strategies proposed in the present work, eventually showing the potential of including immediate data evaluation pipelines into the experimentation loop. A similar approach, however, only focusing on large research facilities, has already been published in ^23^. In the aforementioned study ^23^, it is detailed how to build on the experience gained from comparable measurements from a global database during the ongoing experiment. Eventually, this should help the user to make an immediate decision on how experimental parameters could be adjusted. However, this requires a standardized and error-free database. In the example considered in the present work, this kind of database is not required. From the findings presented here, the following conclusions can be drawn:

### Comparability through standardization

Since the experimentally determined intensities and count rates, respectively, can also be displayed and evaluated in a standardized way over the exposure time (counts per second), the strategy proposed and elaborated can be applied to different spectra from different MPs. The same holds true for measurement sequences with different optimized measuring times, which still can be compared and evaluated in a joint approach.

### Outlook for mappings and grid sizes

The new algorithms proposed can even act as a basis for more complex and, therefore, more powerful algorithms inspired by the field of intelligent experimentation or active experimental design. Here, not only the measurement time can be shortened and utilized more efficiently, but also the next measurement position within the specimen volume or the density and number of measurement points in an iteration can be chosen and assessed during an ongoing measurement series.

### Extending selection strategies

The selection strategy *min_vol* can be further improved using insights from active learning^24^. In active learning, the idea is to select samples such that the model, i.e., the data selector, is improved. Depending on the target parameter, we could develop individual selection strategies that directly optimize these.

### Stopping criterion

In the present study, we have not yet introduced an early stopping criterion. Introducing such a criterion bears the potential to increase the experimentation time savings even further. This can range from defining simple thresholds, e.g., using uncertainties of peak characteristics, to using active learning strategies in combination with automated stopping criteria^24^.

### Intelligent methods

We could apply intelligent methods to use a more sophisticated parameter estimation method instead of fitting a normal distribution model, i.e., Gaussian profiles.

### Overlapping peak evaluation

In addition to the previously used variables for describing a peak profile, the termination conditions or required exposure times can also be linked to allow for a successful evaluation of two overlapping peaks, cf peaks 1 and 9 in present work. The target function that has to be fitted to the spectrum basically consists of a number of different sub-functions. In the simplest case, different Gaussian functions add up for a specific crystalline material. These are supplemented by a background function, which considers the used setup. This function can be characterized in a reference measurement. If polymers are examined, a non-linear polynomial background of a higher order is added to the individual Gaussian functions. Since the target function is always the sum of individual functions, our here-proposed algorithms also offer the possibility to adapt these to the required spectrum. This approach needs to be validated experimentally in future work. Additionally, the use of Mexican-Hat wavelets is a feasible option for peak search and profile analysis. Due to their symmetrical nature, these could be used to identify non-symmetrical peaks and, therefore, to identify potential overlapping peaks. This approach is subject of ongoing work, first promising results obtained for a polyamide specimen are available.

### Human-in-the-loop

In itself, the algorithm is suitable for automated experimentation. Even if automated experiments (without human intervention) are desirable in most cases, integration of a human into the experiment cycle can be useful. Especially in areas where a Bragg peak intensity drops below a certain threshold, human-in-the-loop techniques can be applied. This way, the human could help to optimize the experiment by, e.g., assessing whether a specific measurement point or range requires further investigation.

### Usability

Due to the simplicity of the selection strategies, integrating these into the experimentation loop is thought to be straightforward. Thus, the results and approaches presented in present work bear the potential to become an important standard part of any energy-dispersive XRD experiments. Especially for systems that can communicate with the programming language Python, the code can be used ad-hoc. No previous learning strategy is necessary.

### Transfer to large-scale research facilities

The selection strategies introduced are of highest benefit for laboratory applications, however,can also be applied at large-scale research facilities, such as synchrotrons or high-energy laboratory sources like a liquid metal jet tube, for in situ studies^23,25^.

### Other applications

The data selection strategies within this article can certainly be applied to other experimentation methods. The prerequisites are prior knowledge with respect to the data structure, e.g., the seed peaks for the diffraction profile in our use case. In addition, the exploitation area must be scannable in a flexible manner. Of course, applying these selection strategies is only meaningful for experiments that can be terminated at an earlier point in time.

## Supplementary Information

Supplementary Information 1.

Supplementary Information 2.

Supplementary Information 3.

Supplementary Information 4.

Supplementary Information 5.

Supplementary Information 6.

Supplementary Information 7.

Supplementary Information 8.

Supplementary Information 9.

## Data Availability

The datasets generated and/or analysed in the present study are available using the corresponding DOI ( https://doi.org/10.48662/daks-78 )from the repository of the University of Kassel.

## Code availability

The codes generated in the present study are available using the corresponding DOI (https://doi.org/10.48662/daks-78) from the repository of the University of Kassel.

## References

[1] Weidner, A. In Deformation Processes in TRIP/TWIP Steels, 439, doi: https://doi.org/10.1007/978-3-030-37149-4 (Springer International Publishing, Amsterdam, 2020).

[2] Matlock, D. K. & Speer, J. G. Third Generation of AHSS: Microstructure Design Concepts. In Haldar, A., Suwas, S. & Bhattacharjee, D. (eds.) Microstructure and Texture in Steels, 185–205 (Springer London, London, 2009).

[3] Liehr, A. et al. Experimental Analysis of the Stability of Retained Austenite in a Low-Alloy 42CrSi Steel after Different Quenching and Partitioning Heat Treatments. *Adv. Eng. Mater.* **25**, 16. 10.1002/adem.202300380 (2023).

[4] Richter, J. et al. Metastable CrMnNi steels processed by laser powder bed fusion: experimental assessment of elementary mechanisms contributing to microstructure, properties and residual stress. *Sci. Rep.* **12**. 10.1038/s41598-022-26052-x (2022). 10.1038/s41598-022-26052-x PMC9760645 36529751

[5] Manns, T. & Scholtes, B. Diffraction residual stress analysis in technical components - Status and prospects. *Thin Solid Films* **530**, 53–61. 10.1016/j.tsf.2012.03.064 (2013). 6th Size-Strain International Conference Diffraction analysis of the microstructure of materials.

[6] Breidenstein, B., Heikebrügge, S., Schaumann, P. & Dánekas, C. Influence of the Measurement Parameters on Depth-Resolved Residual Stress Measurements of Deep Rolled Construction Steel using Energy Dispersive X-ray Diffraction. *HTM J. Heat Treatm. Mater.* **75**, 419–432. 10.3139/105.110423 (2020).

[7] Hauk, V. In Structural and Residual Stress Analysis by Nondestructive Methods, 637–640. 10.1016/B978-0-444-82476-9.X5000-2 (Elsevier Science B.V., Amsterdam, 1997).

[8] Stein, H. S. & Gregoire, J. M. Progress and prospects for accelerating materials science with automated and autonomous workflows. *Chem. Sci.* **10**, 9640–9649. 10.1039/c9sc03766g (2019). 32153744 10.1039/c9sc03766g PMC7020936

[9] Stach, E. et al. Autonomous experimentation systems for materials development: A community perspective. *Matter* **4**, 2702–2726. 10.1016/J.MATT.2021.06.036 (2021).

[10] Pollice, R. et al. Data-Driven Strategies for Accelerated Materials Design. *Acc. Chem. Res.* **54**, 849–860. 10.1021/ACS.ACCOUNTS.0C00785 (2021). 33528245 10.1021/acs.accounts.0c00785 PMC7893702

[11] Montoya, J. H. et al. Toward autonomous materials research: Recent progress and future challenges. *Appl. Phys. Rev.* **9**, 011405. 10.1063/5.0076324 (2022).

[12] Kusne, A. G. et al. On-the-fly closed-loop materials discovery via Bayesian active learning. *Nat. Commun.* **11**. 10.1038/S41467-020-19597-W (2020). 10.1038/s41467-020-19597-w PMC7686338 33235197

[13] Lookman, T., Balachandran, P. V., Xue, D., Hogden, J. & Theiler, J. Statistical inference and adaptive design for materials discovery. *Curr. Opin. Solid State Mater. Sci.* **21**, 121–128. 10.1016/j.cossms.2016.10.002 (2017).

[14] Rohr, B. et al. Benchmarking the acceleration of materials discovery by sequential learning. *Chem. Sci.* **11**, 2696–2706. 10.1039/C9SC05999G (2020). 34084328 10.1039/c9sc05999g PMC8157525

[15] Lookman, T., Alexander, F. J. & Bishop, A. R. Perspective: Codesign for materials science: An optimal learning approach. *APL Mater.* **4**, 053501. 10.1063/1.4944627 (2016).

[16] Balachandran, P. V., Xue, D., Theiler, J., Hogden, J. & Lookman, T. Adaptive strategies for materials design using uncertainties. *Sci. Rep.* **6**. 10.1038/srep19660(2016). 10.1038/srep19660 PMC4726355 26792532

[17] Jin, Z., Zhang, Z., Demir, K. & Gu, G. X. Machine Learning for Advanced Additive Manufacturing. *Matter* **3**, 1541–1556. 10.1016/j.matt.2020.08.023 (2020).

[18] Boyce, B. L. & Uchic, M. D. Progress toward autonomous experimental systems for alloy development. *MRS Bull.* **44**, 273–280. 10.1557/MRS.2019.75 (2019).

[19] Noack, M. M. et al. A Kriging-Based Approach to Autonomous Experimentation with Applications to X-Ray Scattering. *Sci. Rep.* **9**, 1–19. 10.1038/s41598-019-48114-3 (2019). 10.1038/s41598-019-48114-3 PMC6694190 31413339

[20] Meier, D. et al. Reconstruction of incomplete X-ray diffraction pole figures of oligocrystalline materials using deep learning. *Sci. Rep.* **13**, 5410. 10.1038/s41598-023-31580-1 (2023). 37012276 10.1038/s41598-023-31580-1 PMC10070271

[21] Dingel, K. et al. AI - Based On The Fly Design of Experiments in Physics and Engineering. In 2021 IEEE International Conference on Autonomic Computing and Self-Organizing Systems Companion (ACSOS-C), 150–153, 10.1109/ACSOS-C52956.2021.00048 (2021).

[22] Liehr, A. et al. Energy Resolved Residual Stress Analysis with Laboratory X-Ray Sources. *HTM J. Heat Treatm. Mater.* **72**, 115–121. 10.3139/105.110316 (2017).

[23] Pithan, L. et al. Closing the loop: autonomous experiments enabled by machine-learning-based online data analysis in synchrotron beamline environments. *J. Synchrotron Radiat.* **30**, 1064–1075. 10.1107/S160057752300749X (2023). 37850560 10.1107/S160057752300749X PMC10624034

[24] Scharei, K. et al. Automated Active Learning with a Robot. *Arch. Data Sci. Ser. A (Online First)* **5**, 16. 10.5445/KSP/1000087327/16(2018).

[25] Sommer, N. et al. High-Throughput Alloy Development Using AdvancedCharacterization Techniques During Directed EnergyDeposition Additive Manufacturing. *Adv. Eng. Mater.* **25**, 16. 10.1002/adem.202300030 (2023).

