# Supplementary material for: Data selection strategies for minimizing measurement time in materials characterization
Source: Sci Rep. 2025 Apr 30;15:15182. doi: 10.1038/s41598-025-96221-1 (PMC12043836; doi:10.1038/s41598-025-96221-1)
Supplement: Supplementary file 1 — Supplementary Information 1. [file 41598_2025_96221_MOESM1_ESM.pdf]

Data selection strategies for minimizing measurement time in complex materials characterization

Alexander Liehr[1,*], Kristina Dingel[2], Daniel Kottke[2], Sebastian Degener[3], David Meier[2,4], Bernhard Sick[2], and Thomas Niendorf[1]

[1]Institute of Materials Engineering, University of Kassel, Kassel, 34121, Germany
[2]Intelligent Embedded Systems, University of Kassel, Kassel, 34121, Germany
[3]Bundesanstalt für Materialforschung und -prüfung, Unter den Eichen 87, 12205 Berlin, Germany
[4]Helmholtz-Zentrum für Materialien und Energie, Hahn-Meitner-Platz 1, 14109 Berlin, Germany
[*]liehr@uni-kassel.de

ABSTRACT

The increasing number of material alloy variants also increases the great importance of the efficiency of well known analysis methods, like for example in the X-Ray diffraction analysis. Particularly in laboratory measurements, where the intensities in diffraction experiments tend to be low, adapting the exposure time to the investigated microstructure is crucial. The counting time is decisive for, e.g., complex texture, phase, and residual stress measurements. Generally, more measurement points lead to more accurate material characterizations. Too short counting times result in poor signal-to-background ratios or too dominant signal noise, making subsequent evaluation more difficult or even impossible. Then, it is necessary to repeat experiments with adjusted, usually significantly longer counting time. To prevent redundant measurements, it is state-of-the-art to use the entire measurement range regardless of whether the investigated points are relevant and contribute to the subsequent materials characterization. This study shows how different selection strategies can decrease energy dispersive diffraction experiment times according to the material's microstructure without losing data quality for subsequent analyses.
All relevant data including the code can be found using the corresponding URL/DOI. This DOI is provided here after acceptance of the manuscript.

Appendix A: Ground Truth Estimation

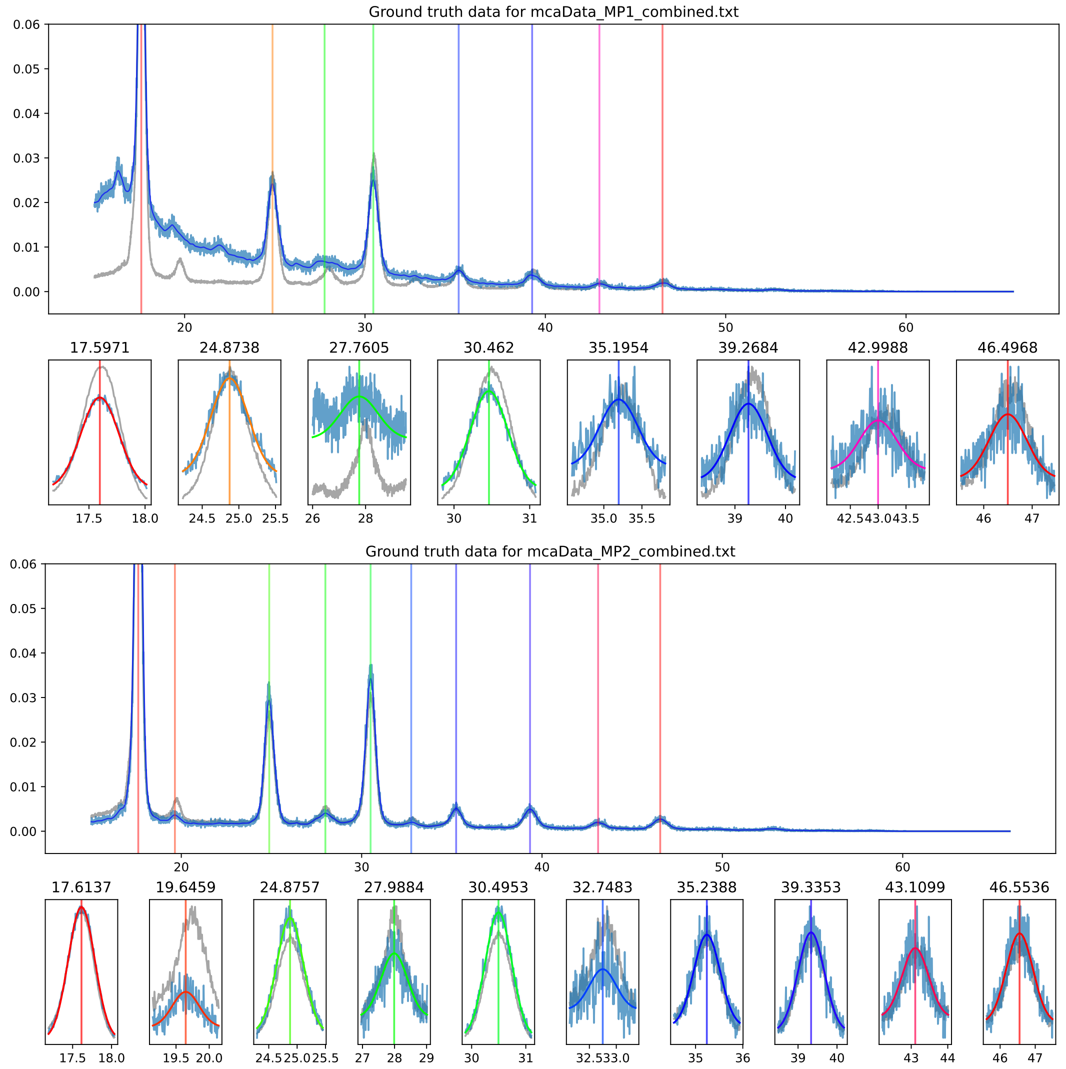

Supplementary Figure 1. Estimation of the ground truth characteristics for all datasets. We fit a normal distribution model (incl. constant bias). From that normal distribution model, we identify the Bragg peak's position, volume, and integral. In gray color, we see the average over all datasets.

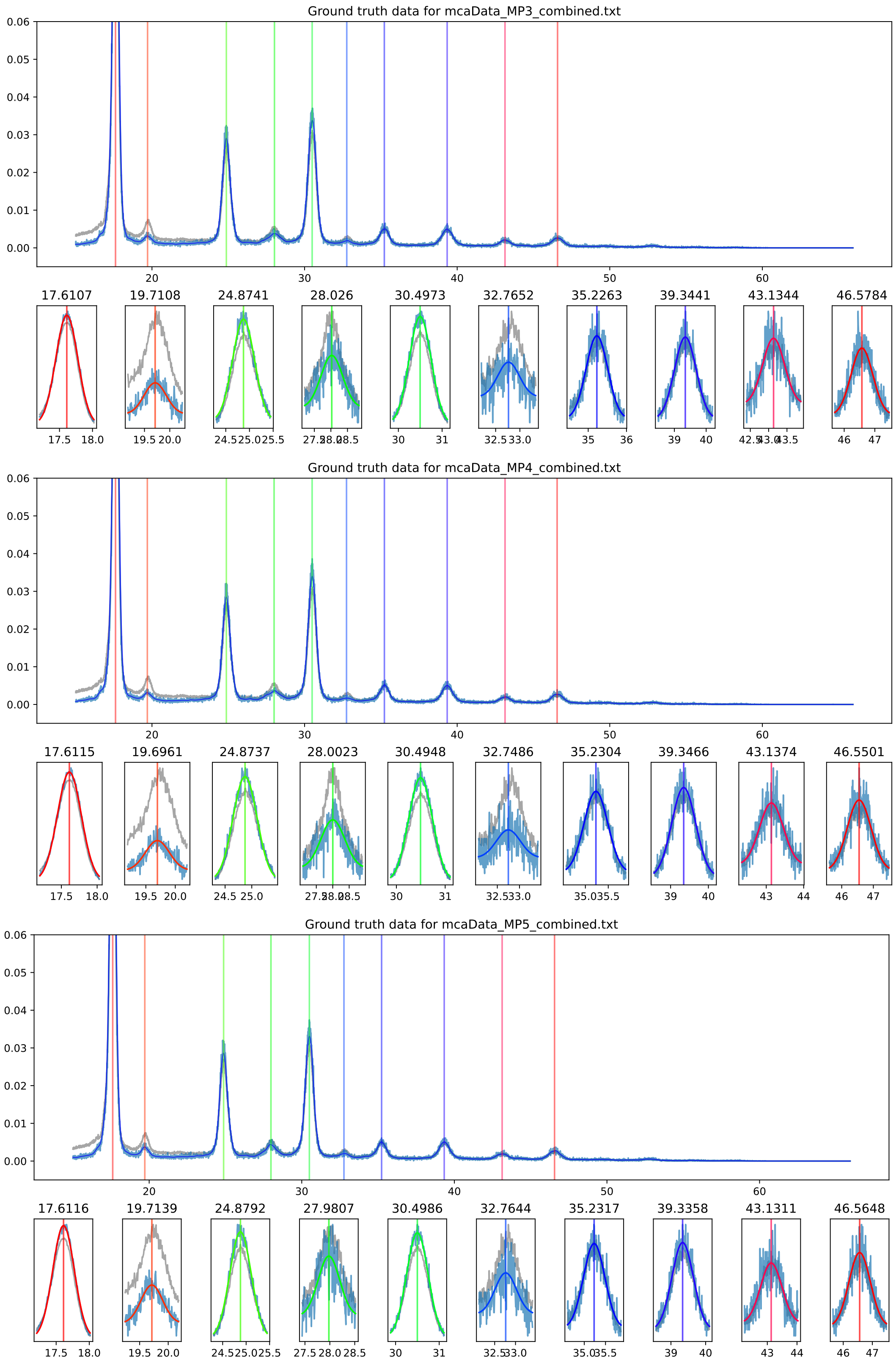

Supplementary Figure 2. Estimation of the ground truth characteristics for all datasets. We fit a normal distribution model (incl. constant bias). From that normal distribution model, we identify the Bragg peak's position, volume, and integral. In gray color, we see the average over all datasets.

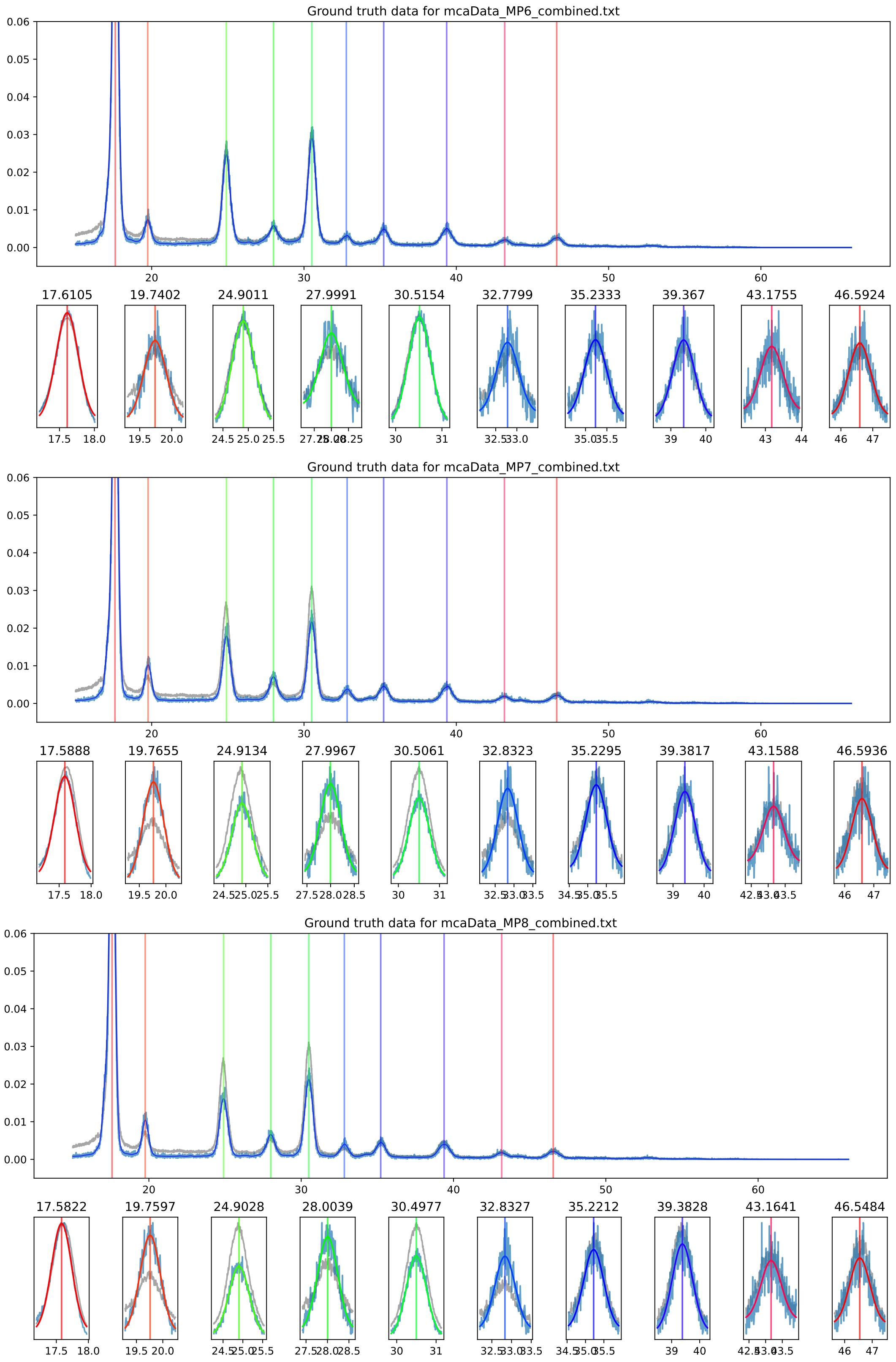

Supplementary Figure 3. Estimation of the ground truth characteristics for all eight datasets. We fit a normal distribution model (incl. constant bias). From that normal distribution model, we identify the Bragg peak's position, volume, and integral. In gray color, we see the average over all datasets.
