# Supplementary figures and images for: Data selection strategies for minimizing measurement time in materials characterization
Source: Sci Rep. 2025 Apr 30;15:15182. doi: 10.1038/s41598-025-96221-1 (PMC12043836; doi:10.1038/s41598-025-96221-1)

Ground truth data for mcaData\_MP1\_combined.txt

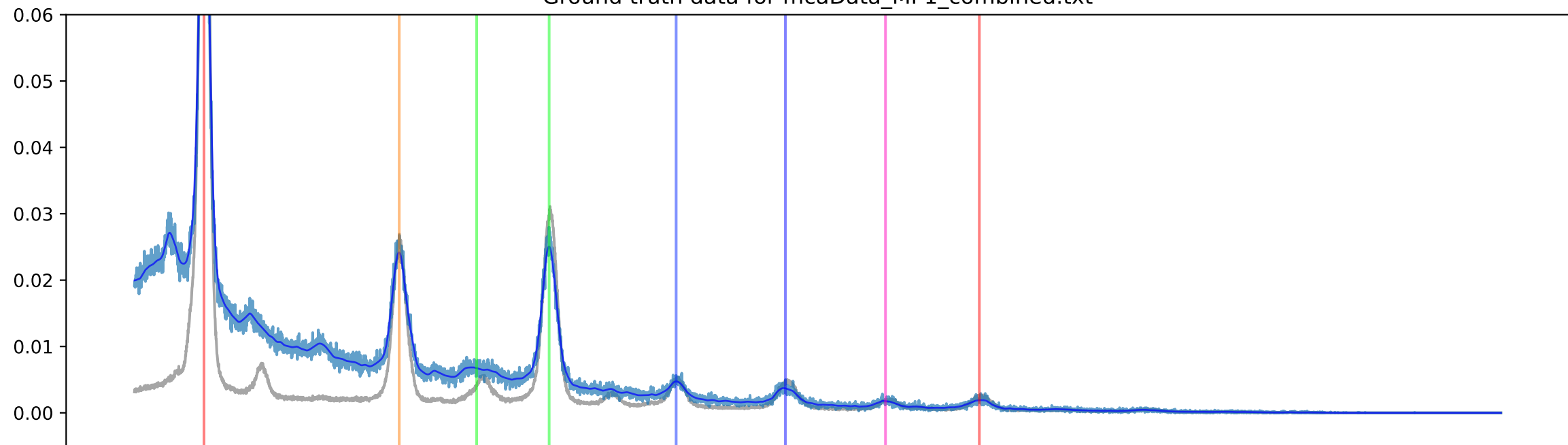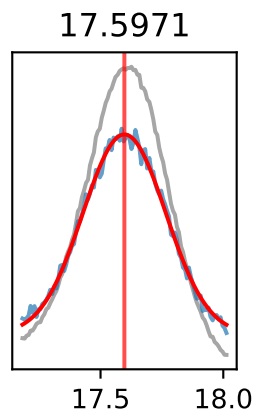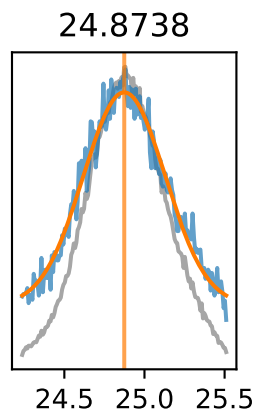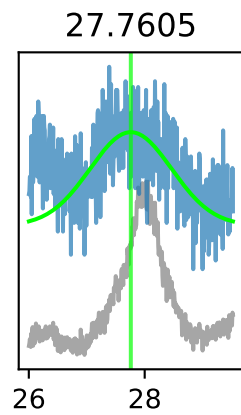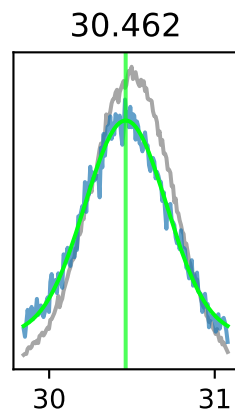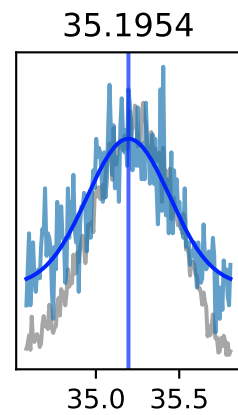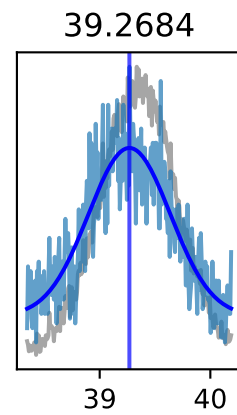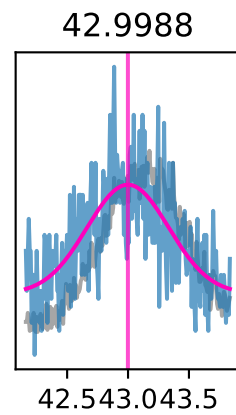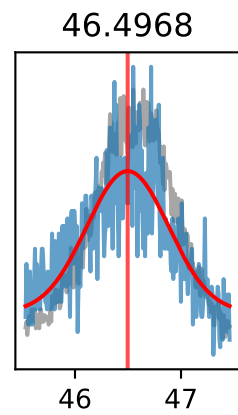

Supplement: Supplementary file 2 — Supplementary Information 2. [file 41598_2025_96221_MOESM2_ESM.pdf]

Ground truth data for mcaData\_MP2\_combined.txt

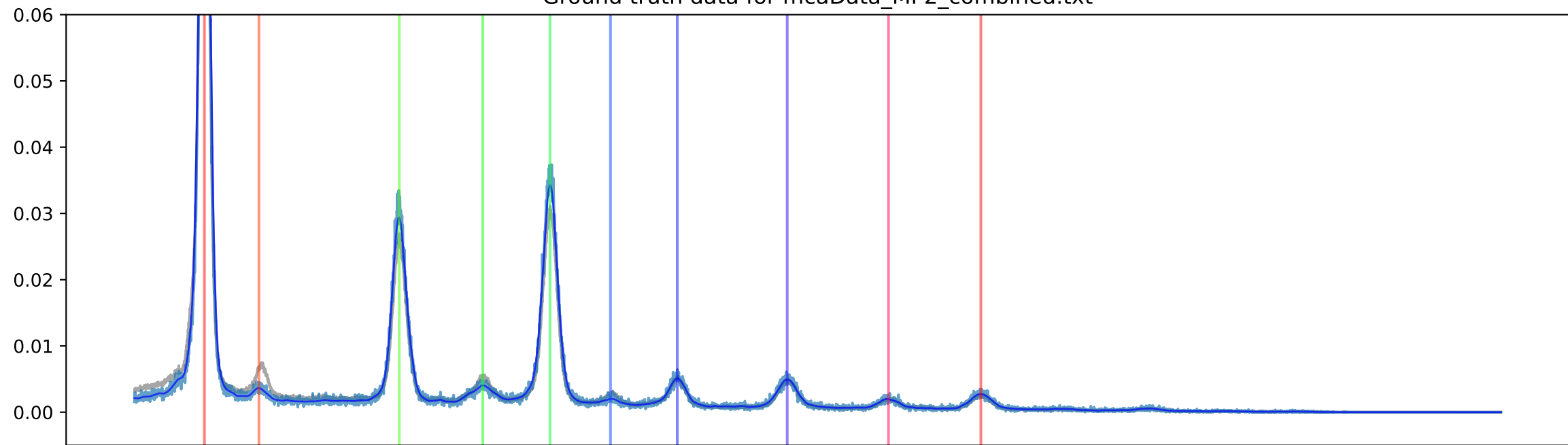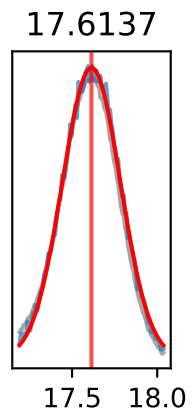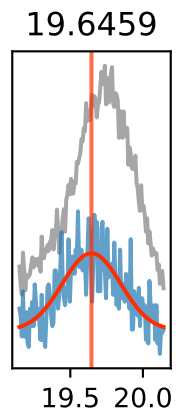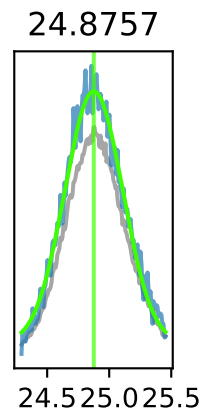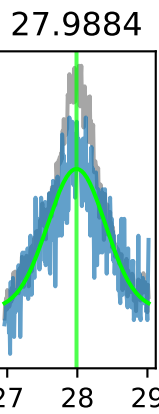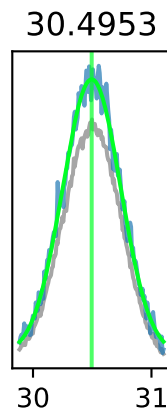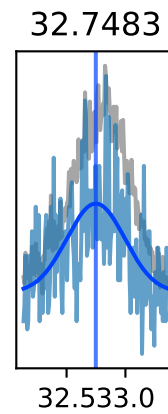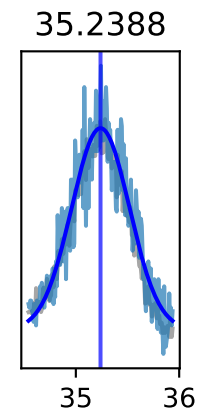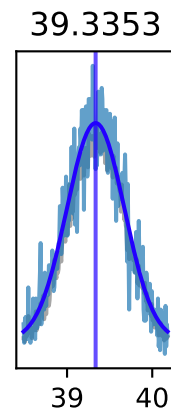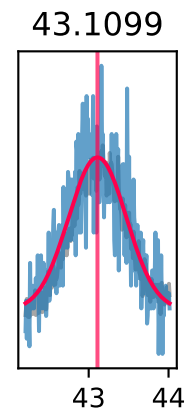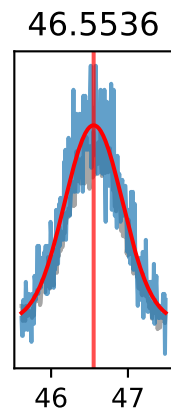

Supplement: Supplementary file 3 — Supplementary Information 3. [file 41598_2025_96221_MOESM3_ESM.pdf]

Ground truth data for mcaData\_MP3\_combined.txt

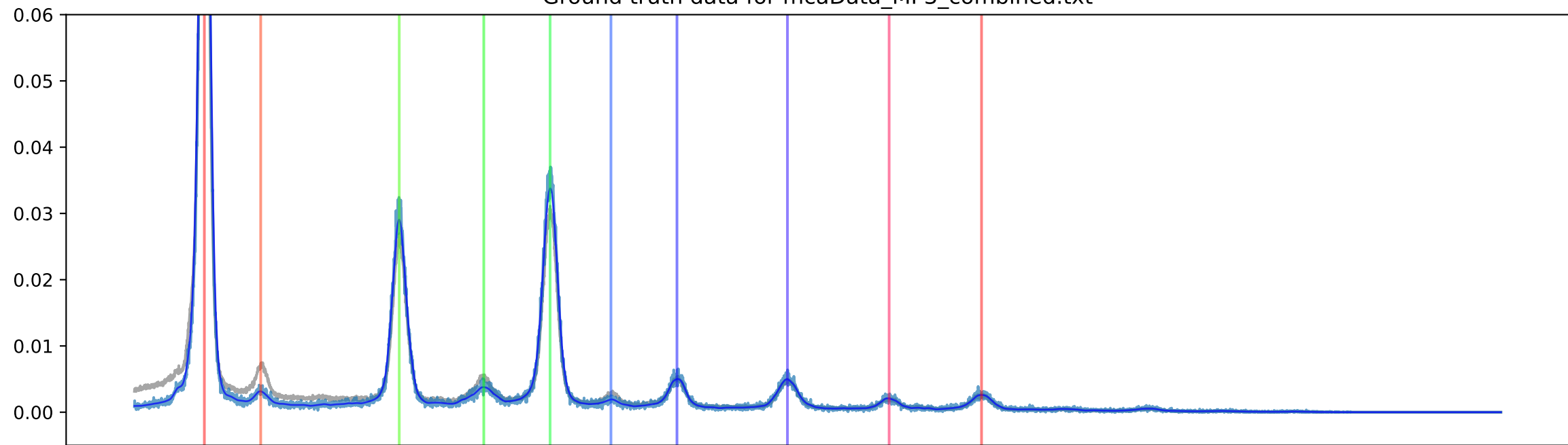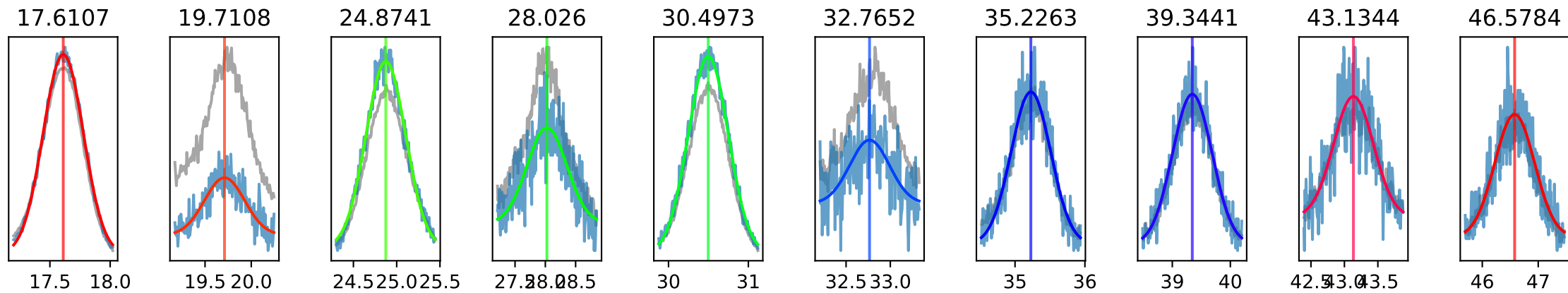

Supplement: Supplementary file 4 — Supplementary Information 4. [file 41598_2025_96221_MOESM4_ESM.pdf]

Ground truth data for mcaData\_MP4\_combined.txt

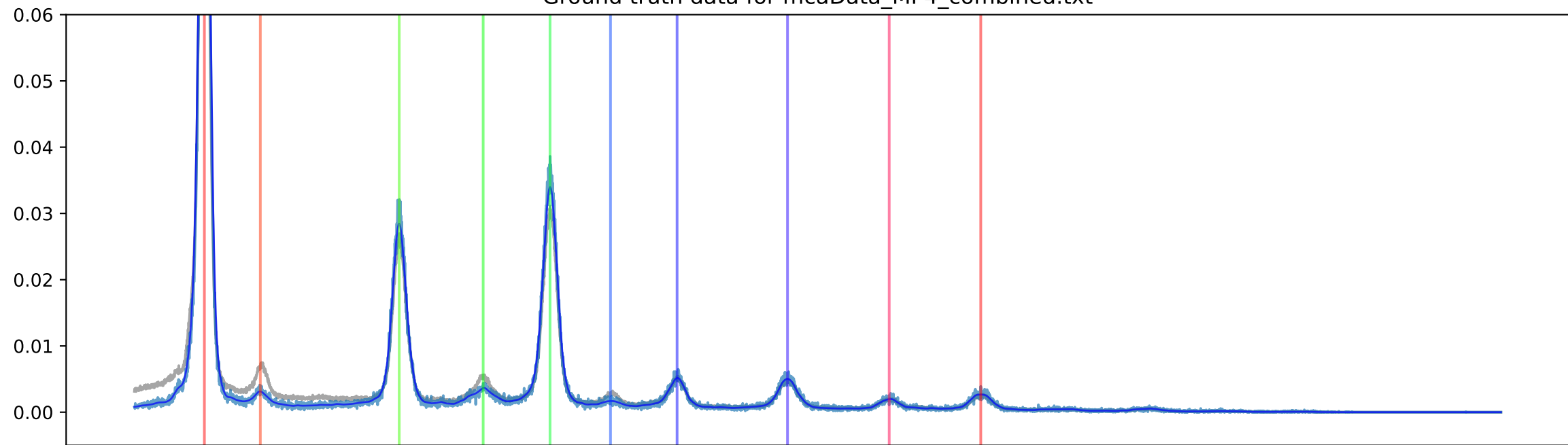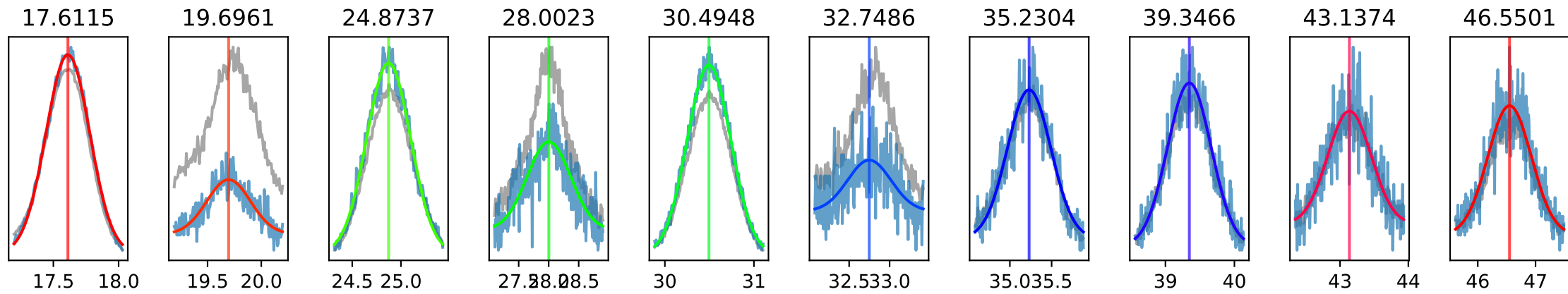

Supplement: Supplementary file 5 — Supplementary Information 5. [file 41598_2025_96221_MOESM5_ESM.pdf]

Ground truth data for mcaData\_MP5\_combined.txt

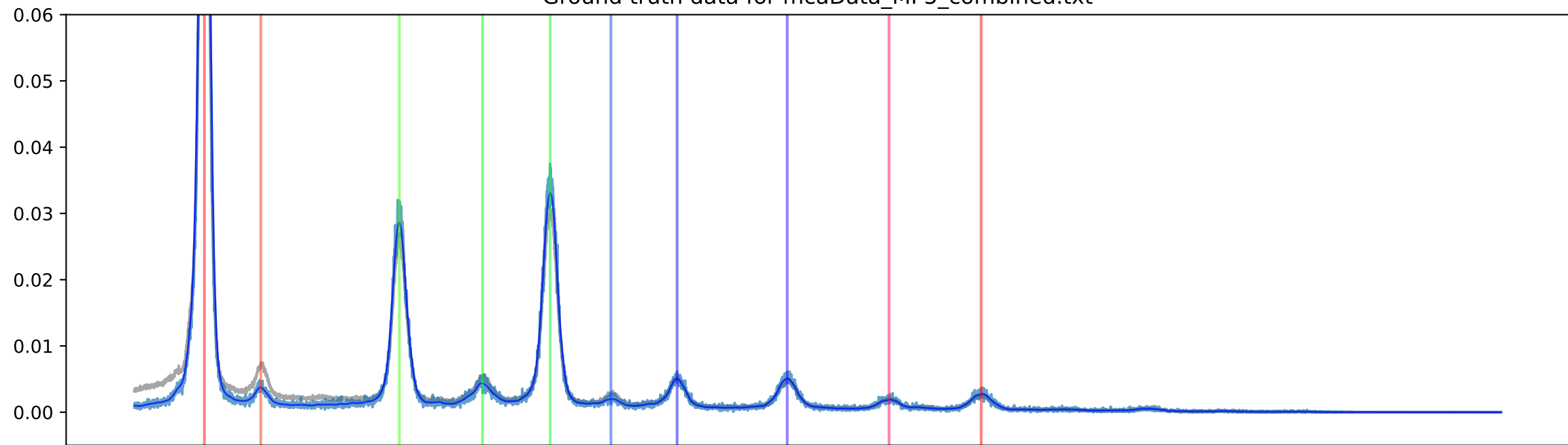

17.6116

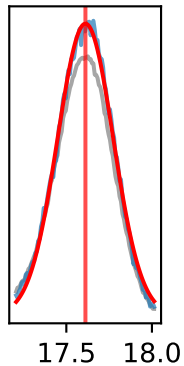

19.7139

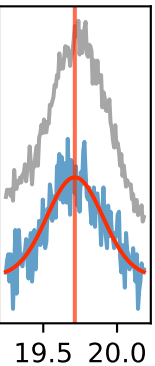

24.8792

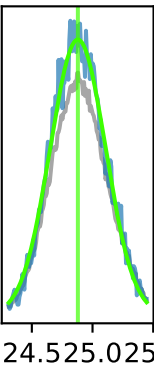

27.9807

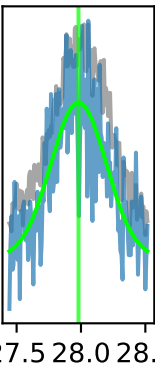

30.4986

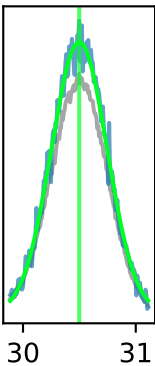

32.7644

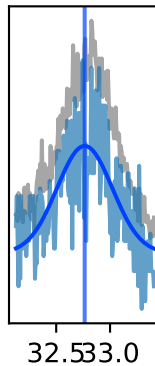

35.2317

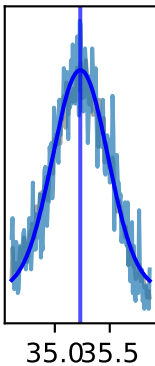

39.3358

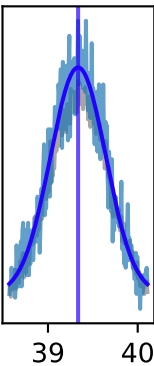

43.1311

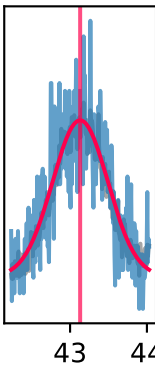

46.5648

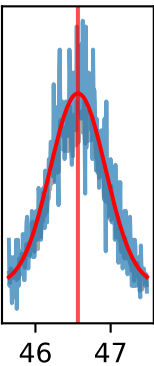

Supplement: Supplementary file 6 — Supplementary Information 6. [file 41598_2025_96221_MOESM6_ESM.pdf]

Ground truth data for mcaData\_MP6\_combined.txt

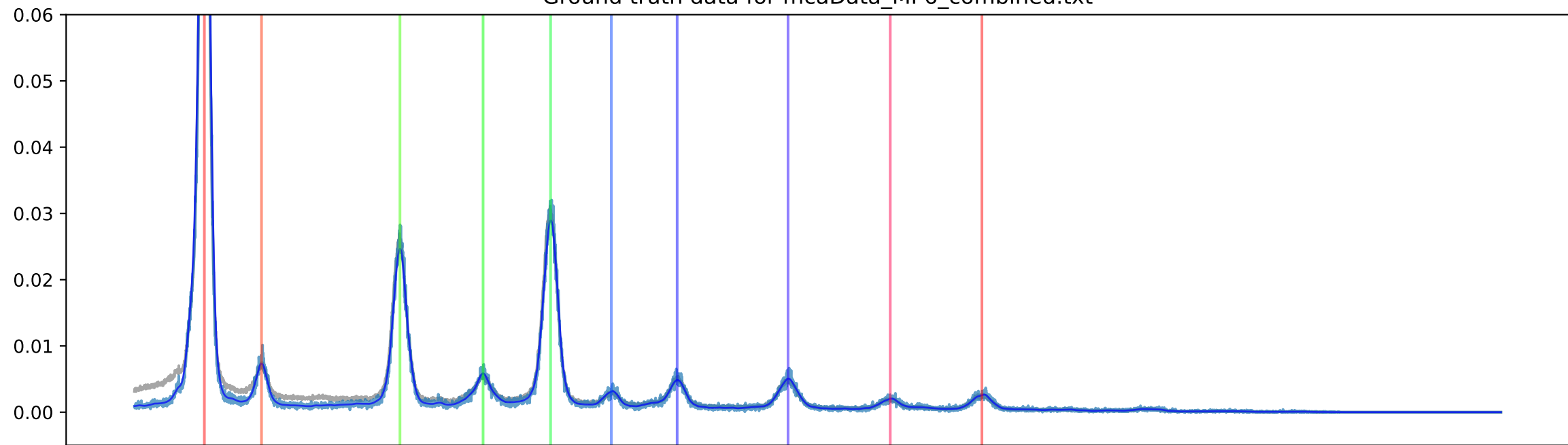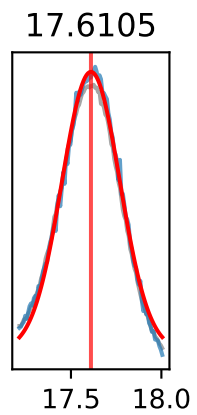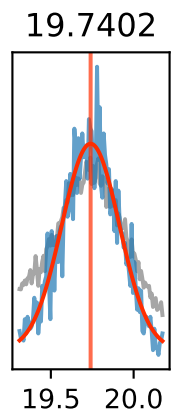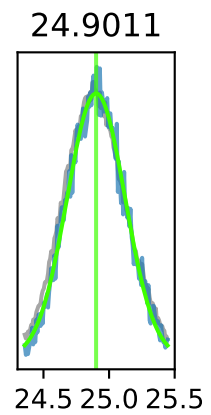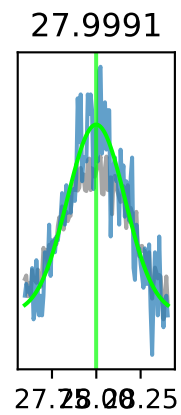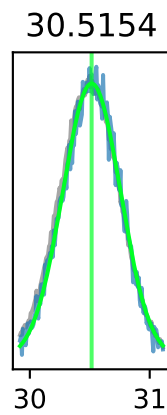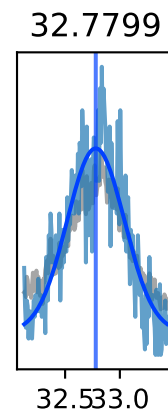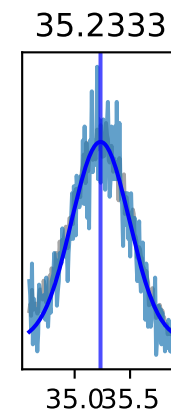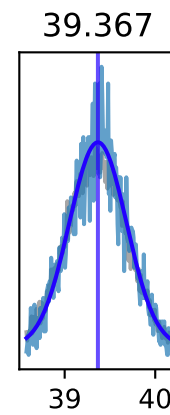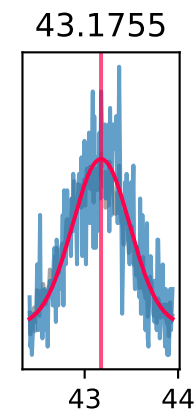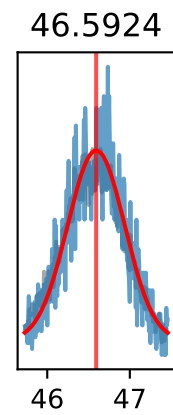

Supplement: Supplementary file 7 — Supplementary Information 7. [file 41598_2025_96221_MOESM7_ESM.pdf]

Ground truth data for mcaData\_MP7\_combined.txt

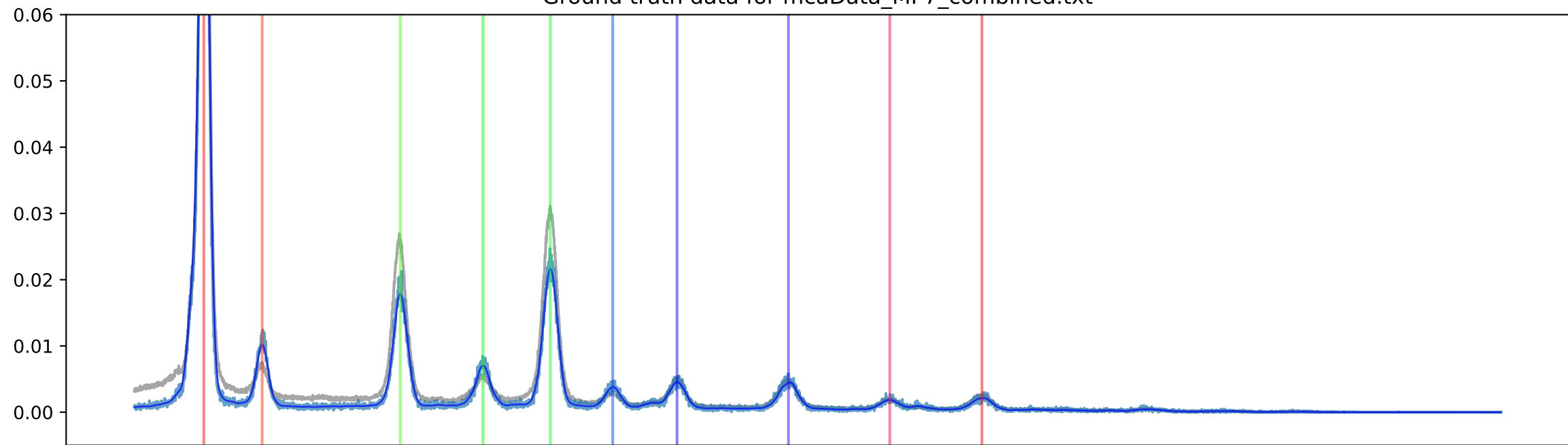

17.5888

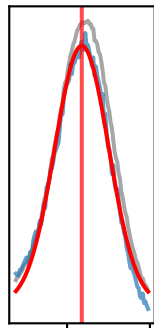

19.7655

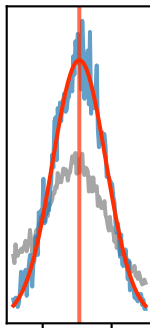

24.9134

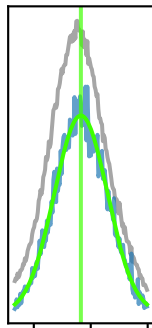

27.9967

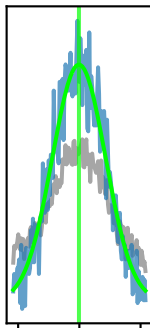

30.5061

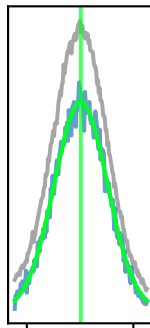

32.8323

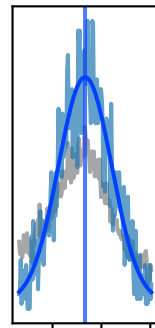

35.2295

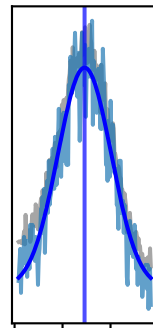

39.3817

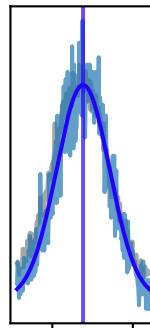

43.1588

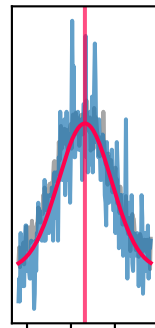

46.5936

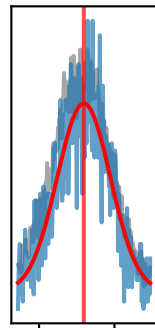

Supplement: Supplementary file 8 — Supplementary Information 8. [file 41598_2025_96221_MOESM8_ESM.pdf]

Ground truth data for mcaData\_MP8\_combined.txt

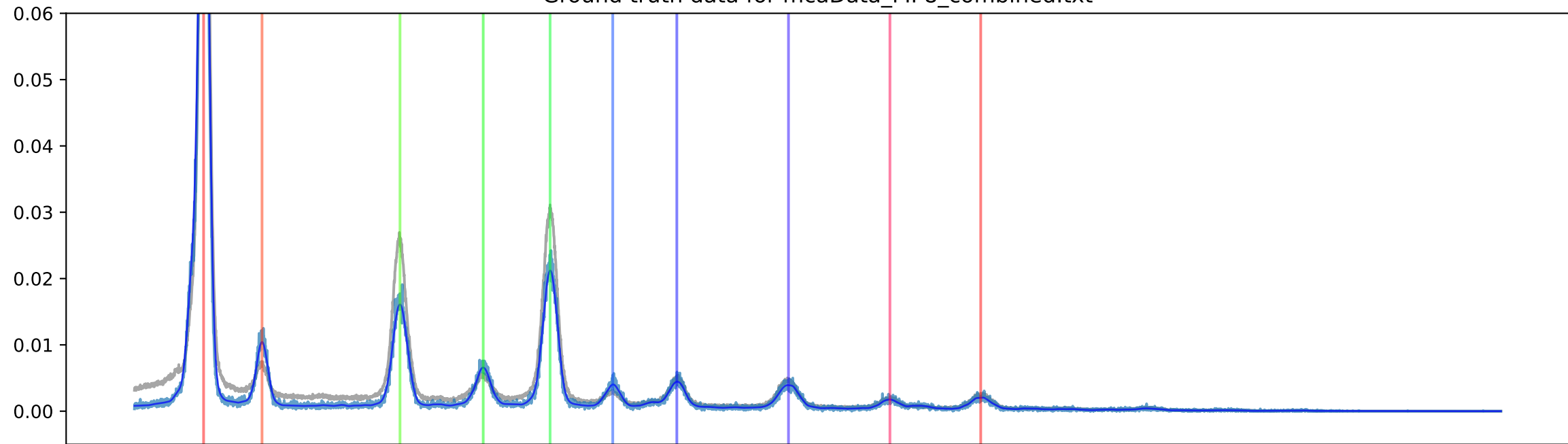

17.5822

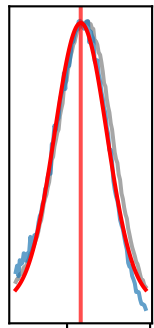

19.7597

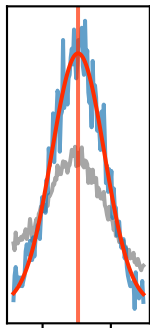

24.9028

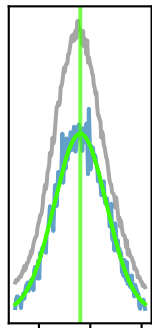

28.0039

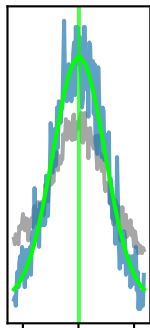

30.4977

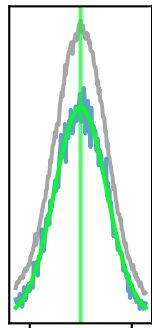

32.8327

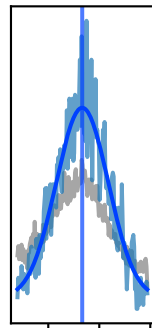

35.2212

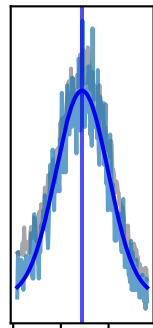

39.3828

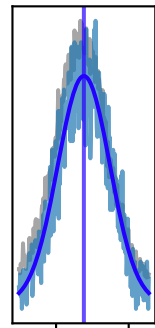

43.1641

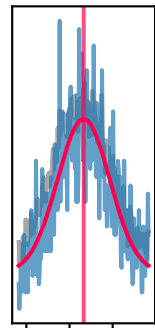

46.5484

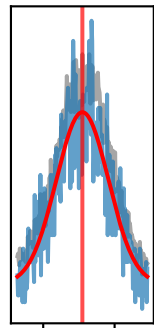

Supplement: Supplementary file 9 — Supplementary Information 9. [file 41598_2025_96221_MOESM9_ESM.pdf]
